# Japanese Perception of Organ Donation and Implications for New Medical Technologies: Quantitative and Qualitative Social Media Analyses

**DOI:** 10.2196/55797

**Published:** 2024-07-19

**Authors:** Xanat Vargas Meza, Masanori Oikawa

**Affiliations:** 1 Institute for the Advanced Study of Human Biology Kyoto University Kyoto Japan; 2 Department of Medical Ethics, Graduate School of Medicine Tohoku University Sendai Japan

**Keywords:** Japan, organ donation, social media, multidimensional analysis, Twitter/X, YouTube

## Abstract

**Background:**

The Rapid Autopsy Program (RAP) is a valuable procedure for studying human biology and diseases such as cancer. However, implementing the RAP in Japan necessitates a thorough understanding of concepts such as good death and the integration of sociocultural aspects. By revising perceptions of organ donation on social media, we bring attention to the challenges associated with implementing new medical research procedures such as the RAP.

**Objective:**

This study aims to examine YouTube and Twitter/X to identify stakeholders, evaluate the quality of organ donation communication, and analyze sociocultural aspects associated with organ donation. Based on our findings, we propose recommendations for the implementation of new medical research procedures.

**Methods:**

Using the term “臓器提供” (organ donation), we collected data from YouTube and Twitter/X, categorizing them into 5 dimensions: time, individuality, place, activity, and relationships. We utilized a scale to evaluate the quality of organ donation information and categorized YouTube videos into 3 groups to analyze their differences using statistical methods. Additionally, we conducted a text-based analysis to explore narratives associated with organ donation.

**Results:**

Most YouTube videos were uploaded in 2021 (189/638, 29.6%) and 2022 (165/638, 25.9%), while tweets about organ donation peaked between 2019 and 2022. Citizens (184/770, 23.9%), media (170/770, 22.0%), and unknown actors (121/770, 15.7%) were the primary uploaders of videos on organ donation. In a sample of average retweeted and liked tweets, citizens accounted for the majority of identified users (64/91, 70%, and 65/95, 68%, respectively). Regarding Japanese regions, there were numerous information videos about organ donation in Hokkaido (F2.46,147.74=–5.28, *P*=.005) and Kyushu and Okinawa (F2.46,147.74=–5.28, *P*=.005). On Twitter/X, Japan and China were the most frequently mentioned countries in relation to organ donation discussions. Information videos often focused on themes such as borrowed life and calls to register as donors, whereas videos categorized as no information and misinformation frequently included accusations of organ trafficking, often propagated by Chinese-American media. Tweets primarily centered around statements of donation intention and discussions about family consent. The majority of video hyperlinks directed users to YouTube and Twitter/X platforms, while Twitter/X hyperlinks predominantly led to news reports from Japanese media outlets.

**Conclusions:**

There is significant potential to implement new medical research procedures such as the RAP in Japan. Recommendations include conceptualizing research data as borrowed data, implementing horizontally diversified management of donation programs, and addressing issues related to science misinformation and popular culture trends.

## Introduction

### A New Medical Research Paradigm in Japan

Japan faces the challenge of a super-aging society, with cancer being the leading cause of death as of 2021 [1]. Therefore, Japanese researchers must prioritize cancer prevention and treatment efforts. However, recent advancements in gene sequencing have highlighted the value of the Rapid Autopsy Program (RAP) in North America [2]. The RAP involves the quick removal of tissues within hours after death, with the prior consent of potential donors [3]. This technique is particularly effective for cancer research. One of the main issues with implementing the RAP in Japan is the cultural concept of a “good death.” This involves understanding the diagnosis and treatment options, deciding on the place of treatment or rest, and preparing for the outcomes of death, as agreed upon by the patient and their relatives [4].

These contradictions significantly impact medical research and end-of-life care. As discussing death can be challenging, other forms of medical intervention need to be analyzed and compared with the RAP. This approach aims to develop medical research procedures that respect human dignity while incorporating Japan’s sociocultural aspects. The process of organ donation shares several overlapping characteristics: surgical intervention in an anonymous donor’s body, at least one anonymous recipient of the benefits, a donation coordinator, and the involvement of medical professionals and governmental institutions. This is why, in this study, we review public perceptions of organ donation in Japan, aiming to provide recommendations for implementing new medical research procedures closely related to end-of-life care, such as the RAP.

### Organ Donation in Japan

In Japan, the first law regarding organ donation was the Law for the Transplantation of Kidneys and Corneas, enacted in 1979. This was followed by the establishment of the Kitasato University Hospital Bone Bank and further legislation in 1997, which recognized brain death exclusively in the context of organ transplantation [5]. Currently, the organization overseeing organ donation in Japan is the Japan Organ Transplantation Network (JOTN). According to the JOTN, in 2000, 93% (71/76) of donation cases occurred after heart death and 7% (5/76) occurred after brain death. By contrast, by 2020, 88% (68/77) of donation cases occurred after brain death and 12% (9/77) occurred after heart death [6]. This shift resulted from the legal change in 2010, which allowed organ donation following brain death with the family’s consent. Although the total number of cases remained similar, the average number of organs handled per donor was 6.8, which is considered high [7]. Furthermore, Japan is among the global leaders in living donor organ donations, particularly excelling in kidney donation and transplantation [8].

This may be partly attributed to the significance of kinship within Japanese norms. Ideally, societal systems should not be entirely heteronomous (governed solely by rules implemented by experts and institutions) or entirely autonomous (governed solely by traditional cultural practices), as these extremes can lead to authoritarianism. Instead, a desirable approach involves integrating multiple stakeholders collaborating for the common good. Fuse [9] refers to this social dynamic as *kyosei* (共生, living together), which involves acknowledging inequalities and subordination, respecting heterogeneity, and accepting conflict.

Age, religion, and trust in medical care were considered relevant factors affecting organ donation. Confucianism was found to be more influential than brain death in some contexts, whereas the opposite was observed when donating organs from relatives [10]. A study among medical staff and the public revealed that both groups believe families are reluctant to damage the body or cause additional suffering to the patient and that autopsy raises suspicion and may lead to legal accusations of medical errors [11]. Yasuoka [12] provided a detailed account of attitudes toward organ donation among various stakeholders including medical professionals, donors, recipients, relatives, and coordinators. The study identified skepticism toward narratives from the JOTN and highlighted a challenging environment that frequently led coordinators to resign.

An internet-based survey of medical professionals and the public revealed that 25.4% of the public and 82.3% of staff, particularly cardiothoracic surgeons, supported organ and tissue donation [13]. Additionally, a report on workshops for kidney transplant coordinators, which included role-playing, showed that donation-related knowledge improved after 3 months [14]. An online survey among citizens indicated that while living organ donation was preferred over donation after brain death, the preference shifted when participants considered themselves in a position to donate and receive organs, with donation after brain death being more favored over living donation [15]. Associations and the media were also identified as significant promoters of donation [16,17].

Akabayashi [18] argued that Confucianism, particularly through the principle of filial piety, posed a barrier to organ donation. A survey among medical staff revealed that knowledge about organ donation was not correlated with becoming a registered organ donor, but rather with the willingness to become one [19]. A long-term survey among medical students highlighted an incomplete understanding of the organ transplantation law and the primary reason cited for becoming an organ donor was “to help others” [20]. A literature review of nursing students’ attitudes found that interest in organ donation correlated with an increase in donor cards [21]. The Kitasato Bone Bank ceased donations until protocols for COVID-19 screening were implemented [22], suggesting a potential impact on other organ and tissue donation processes. Additionally, a study conducted at a Baptist hospital reported that religion appeared to provide comfort and relief for donor relatives [23].

### Knowledge Gaps

Based on the reviewed studies, it is estimated that individuals with more medical training and experience with organ donation tend to have more positive attitudes toward donation. However, the role of religion remains unclear, as studies have often not operationalized or disclosed specific religious arguments. While some evidence has indicated relief for donor relatives through Baptism, it remains unclear whether similar effects are present in other religions in Japan. Additionally, only 3 studies [12,16,17] specifically focused on narratives and their adoption by individuals. In summary, most studies have focused on the knowledge, attitudes, and training of medical professionals regarding organ donation. By contrast, fewer studies have addressed donors, recipients, patients, their families, and coordinators, and even less is known about the roles of the media and the general public. Therefore, there is a need to explore attitudes toward organ donation among various stakeholders, considering their interactions and influences on each other.

Given that Japanese individuals are notably active on social media platforms, there are ample opportunities for encountering information about organ donation. Those interested in the topic may engage in consulting, discussing, and sharing such information more openly compared with traditional one-on-one interview formats. While this is a prevalent method in reviewed studies, supplementing it with network analysis methods applied to contemporary social media usage could provide deeper insights.

### Study Objectives

We have formulated the following objectives:

- Identify contributors to the organ donation discourse on Japanese social media.
- Evaluate the quality of the organ donation discourse among these contributors.
- Identify sociocultural aspects associated with organ donation and provide recommendations for the implementation of new medical research procedures based on the findings.

## Methods

### Data Collection

In Japan, YouTube and Twitter/X are among the top 5 most visited social networking sites [24,25]. Therefore, we considered these platforms for data collection in 2 formats: videos on YouTube and tweets on Twitter/X. We used the search term “臓器提供” (organ donation) as our query for gathering relevant content.

We extracted video data from YouTube using YouTube Data Tools [26], utilizing 2 modules that interface with the YouTube application programming interface. The Video List module enabled us to retrieve a list of videos along with related information such as publication date, title, description, duration, number of views, and number of comments. We verified the video titles and watched the videos to identify those related to postmortem organ and tissue donation, resulting in 638 videos out of the initial 2166. Using identifiers obtained from the Video List module (the last part of the video hyperlink), we utilized the Video Comments module to extract comments associated with these videos. Regarding Twitter/X, we requested tweets from the company Tweet Binder [27], which provided us with a data set of 133,434 tweets. As the KH Coder software [28] used for analysis can handle up to 100,000 tweets at a time, we extracted a sample of that size for further analysis, as detailed in the following section.

### Classification of Data

**Figure 1:**
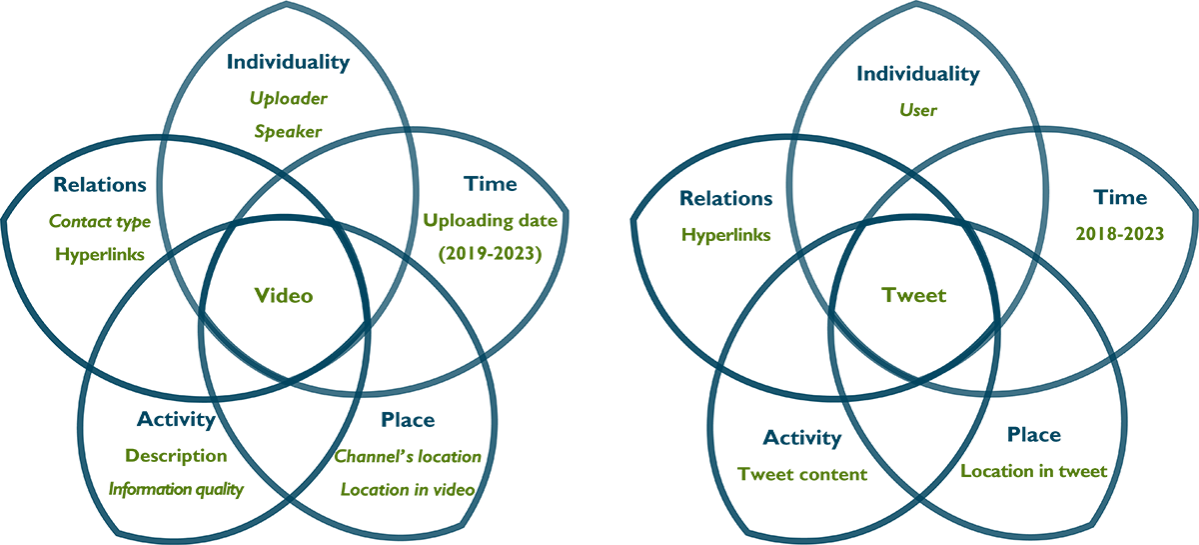
Data model of social media entities.

Based on Vargas Meza and Yamanaka [29] and Vargas Meza and Park [30], we analyzed social media data by considering multiple actors and other factors in line with *kyosei,* as illustrated in Figure 1. We examined YouTube videos (left side of Figure 1) and tweets (right side of Figure 1) as the entities under study. We extracted factors written in nonitalics directly from the data, while factors in italics were verified by XVM.

The 5 dimensions of the entities were operationalized as detailed in Textbox 1.

**Operationalization of the 5 dimensions of the entities.**

1. Time

Refers to the temporal aspect of the YouTube videos and tweets. The upload dates of the YouTube videos ranged from January 2019 to May 2023, while the dates of the tweets spanned from September 2018 to July 2023.

2. Individuality

We referred to the “About” page of the YouTube channel to identify uploaders, and speakers were identified as individuals who appeared, spoke, or whose words were conveyed in the video. On Twitter/X, we utilized a randomizer [31] to select a sample of 100 users with average retweets and likes. Additionally, we analyzed the authors of the top 10 most liked and retweeted tweets and categorized them according to Multimedia Appendix 1.

3. Place

We located the information on the channel’s “About” page and within the YouTube video itself. On Twitter/X, we identified the location mentioned within the tweet’s content. We categorized countries and regions using a place classification scheme (Multimedia Appendix 2).

4. Activity

For the YouTube videos, we utilized the video description and an Information Quality score (Textbox 2) based on [32,33]. The Information Quality score included 8 items formulated as questions about the video content, with a maximum possible score of 7. The final item indicated misinformation and disinformation, which, if present, received a score of –1. Misinformation and disinformation were defined as unreliable, false, deceptive, or heavily politically charged medical information [34]. A researcher (XVM) who specialized in social media and public health verified the items while watching the videos, and a second researcher (MO) who specialized in bioethics and public health verified the same items in 122 out of 638 videos (19.1%). Their agreement per item was 0.91, 0.81, 0.85, 0.91, 0.83, 0.92, 0.97, and 0.76. Despite the moderately low agreement on the last item, we opted to use the coding from the first researcher. For Twitter/X, we considered the most frequently used words in the tweets.

5. Relations

For the YouTube videos, we examined hyperlinks and the type of contact information (eg, blog, email, social networking site, web page) provided in the video descriptions. For the tweets, we considered the hyperlinks included in the tweets.

**Organ Donation Information Quality (ODIQ).**

Does the video:

- contain a medically appropriate definition of brain death?
- contain several definitions of death?
- mention at least two organs that can be donated?
- mention the organ donation card?
- mention other options for donation statements?
- mention the donation process?
- mention it is free of charge for medical procedures for the donor?
- contain misinformation or disinformation?

### Data Analysis

We used a combination of quantitative and qualitative methods to analyze the data. On YouTube, the videos were classified into 3 categories based on their Organ Donation Information Quality (ODIQ) scores: misinformation (videos containing misinformation or disinformation), no information (videos with an ODIQ score of 0), and information (all other videos). We used this classification because misinformation videos often included some information alongside misleading material.

We quantified the numerical results based on our research model (Figure 1) by conducting a 1-way ANOVA for parametric variables (eg, published year, uploaders, speakers, country, place, contact type, consent type, media format, religion, and narratives). For nonparametric variables (eg, duration and views), we used a Kruskal-Wallis test to uncover differences among video groups. A significance threshold of *P*<.01 was considered. Post hoc tests (Multimedia Appendix 3) were conducted using SPSS version 29.0.10 (IBM Corporation) [35].

We analyzed the narratives associated with organ donation as found in YouTube video descriptions and comments, as well as in the content of tweets, using both quantitative and qualitative techniques. Text data were processed using KH Coder version 3 [28] to calculate word frequencies. KH Coder can summarize frequent terms in Japanese and their relationships with other terms, generating co-occurrence networks. In this study, the networks were represented as undirected and unipartite, calculated using the overlap coefficient [36]. The 60 strongest co-occurrences were depicted as network edges.

We conducted a qualitative analysis by annotating and observing videos, following guidelines from Park et al [37], and included additional narratives identified in our previous study (Multimedia Appendix 4; also see [12,17,18,20]). A researcher (XVM) counted these narratives, applying a threshold of at least 17 out of 638 videos (2.7%). We examined the top 10 YouTube videos based on views and comments to further validate narratives and assess the quality of information. Additionally, we analyzed 7 videos that discussed donation for research to closely compare scenarios involving new research procedures. Furthermore, we focused on frequent terms identified through text analysis and reviewed corresponding tweets related to organ donation.

### Ethical Considerations

This study was deemed exempt from ethical review by the Medical Board of Kyoto University because it involved analysis of social media records and did not use human data beyond measuring internet activity. The data analyzed did not contain information that could identify specific individuals, and any findings related to medical conditions were reported anonymously.

## Results

### Time Dimension in Japanese Social Media

**Figure 2:**
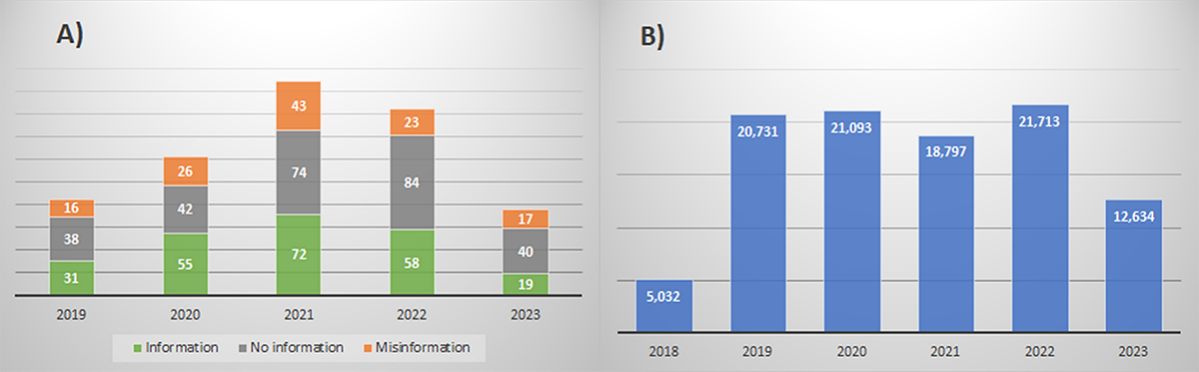
(A) YouTube videos and (B) tweets per year.

Figure 2 illustrates the quantity of YouTube videos from 2019 to 2023 (Figure 2A) and tweets from 2018 to 2023 (Figure 2B). YouTube videos peaked in 2021 (189/638, 29.6%) and 2022 (165/638, 25.9%), with no significant difference (*P*=.05) between the groups. On Twitter/X, mentions of organ donation peaked in 2022 (21,713/100,000, 21.71%), 2020 (21,093/100,000, 21.09%), and 2019 (20,731/100,000, 20.73%). The topic of organ donation evidently peaked in 2022 on both social media platforms.

### Individuality Dimension: Actors in Japanese Social Media

Table 1 presents overlapping actors identified in YouTube videos. Citizens (184/770, 23.9%), media (170/770, 22.0%), and unknown actors (121/770, 15.7%) uploaded the majority of videos about organ donation. A 1-way ANOVA revealed significant differences among the following actors: doctors (*F*_0.92,34.81_=–8.41, *P*<.001), other medical staff (*F*_0.28,14.36_=–0.14, *P*=.002), associations (*F*_1.23,71.70_=–5.45, *P*=.004), citizens (*F*_3.20,127.72_=–7.97, *P*<.001), media (*F*_3.12,121.58_=–8.14, *P*<.001), religion (*F*_2.64,70.29_=–11.93, *P*<.001), and education (*F*_0.63,24.30_=–8.32, *P*<.001). The Bonferroni tests detailed in Multimedia Appendix 3 indicated differences in the means of doctors between information and misinformation (95% CI 0.04-0.16, *P*<.001), and information and no information videos (95% CI 0.01-0.11, *P*=.007). For other medical staff, there was a significant difference between information and no information videos (95% CI 0.01-0.08, *P*=.003), with a moderate difference also observed between information and misinformation videos (95% CI 0.00-0.08, *P*=.03).

Other differences were the means of associations between misinformation and information videos (95% CI –0.21 to –0.03, *P*=.003); means of citizens between misinformation and no information videos (95% CI 0.06-0.29, *P*=.001) and between misinformation and information videos (95% CI 0.06-0.30, *P*=.001); means of media between no information and information videos (95% CI 0.06-0.25, *P*<.001); means of religious actors between misinformation and no information (95% CI 0.03-0.20, *P*=.004) and misinformation and information (95% CI 0.09-0.27, *P*<.001); and means of educational actors between information and no information videos (95% CI 0.03-0.11, *P*<.001).

Regarding speakers, citizens (343/2181, 15.73%) were mostly portrayed in YouTube videos, followed by doctors (272/2181, 12.47%). We observed differences in doctors (*F*_2.80,153.08_=–5.81, *P*=.003), other medical staff (*F*_0.80,46.95_=–5.46, *P*=.004), patients (*F*_2.18,79.37_=–8.72, *P*<.001), associations (*F*_2.78,126.43_=–6.99, *P*=.001), citizens (*F*_2.86,155.73_=–5.83, *P*=.003), the government (*F*_5.16,121.83_=–13.46, *P*<.001), the media (*F*_3.24,135.52_=–7.59, *P*=.001), and religious actors (*F*_4.09,90.17_=–14.42, *P*<.001).

**Table 1:** Actors related to organ donation in videos based on Organ Donation Information Quality.

Actor	IU^a^ (n=281), n	NU^b^ (n=364), n	MU^c^ (n=152), n	TU^d^ (n=770), n	IS^e^ (n=764), n	NS^f^ (n=907), n	MS^g^ (n=510), n	TS^h^ (n=2181), n
Doctor	25^i^	12	1	38	106	99^i^	66^i^	271
Medical student	0	0	0	0	4	3	1	8
Nurse	3	1	0	4	34	42	21	97
Other medical staff	12^i^	2	1	15	30^i^	14^i^	8	52
Donor	2	1	0	3	67	91	55	213
Recipient	3	7	0	10	47	52	26	125
Donor relative	0	0	0	0	33	24	11	68
Recipient relative	0	0	0	0	16	22	9	47
Other patient	4	10	1	15	26	60^i^	10	96
Other patient relative	2	0	0	2	21	36	6	63
Association	40^i^	38	6^i^	84	86^i^	61	33^i^	180
Citizen	59	71	54^i^	184	116	143	84^i^	343
Government	9	4	1	14	52	66	57^i^	175
Media	42	93^i^	35	170	54^i^	99	51	204
Religion	16	37	31^i^	84	27	46	42^i^	115
Education	16^i^	37^i^	0	26	37	34	25	96
Unknown	48	51	22	121	8	15	5	28

^a^IU: information uploader.

^b^NU: no information uploader.

^c^MU: misinformation uploader.

^d^TU: total uploader.

^e^IS: information speaker.

^f^NS: no information speaker.

^g^MS: misinformation speaker.

^h^TS: total speaker.

^i^Statistically significant differences.

Bonferroni tests revealed significant differences in the mean representation of various speaker categories across YouTube videos: doctors differed between misinformation and no information (95% CI 0.04-0.30, *P*=.004); other medical staff differed between no information and information (95% CI –0.08 to 0.06, *P*=.004); patients differed between no information and misinformation (95% CI 0.04-0.23, *P*=.001) and between no information and information (95% CI 0.03-0.18, *P*=.003); associations differed between information and no information (95% CI 0.05-0.24, *P*=.001); citizens differed between misinformation and no information (95% CI 0.03-0.29, *P*=.01) and between misinformation and information (95% CI 0.05-0.31, *P*=.004); the government differed between misinformation and no information (95% CI 0.11-0.33, *P*<.001) and between misinformation and information (95% CI 0.12-0.35, *P*<.001); media differed between information and misinformation (95% CI –0.30 to –0.06, *P*=.002) and between information and no information (95% CI –0.22 to –0.03, *P*=.006); and religious actors differed between misinformation and no information (95% CI –0.22 to –0.03, *P*<.001) and between misinformation and information (95% CI 0.12-0.32, *P*<.001).

A sample of 91 users who tweeted 100 tweets, including 2 retweets, revealed that 64 (70%) were identified as citizens. Similarly, among 95 users who generated 100 tweets with 8 likes, 65 (68%) were identified as citizens. Two registered donors were found among the citizens in each category. Additionally, users with the most likes generally expressed support for organ donation, including a doctor and a politician. However, another doctor, unrelated to transplantation, expressed a negative stance (Tables 2 and 3). Among users with the most retweets, half expressed support, including a doctor and a politician, while another doctor’s stance was unclear, and yet another was not in favor.

**Table 2:** Top users (and their likes) identified on Twitter/X.

Username	Likes, n	Type	In favor of donation?
@aaaaa	82,004	Citizen	Yes
@bbbbb	37,095	Citizen	Unclear
@ccccc	27,028	Doctor	No
@ddddd	24,162	Unknown	Yes
@eeeee	18,709	Citizen	Yes
@nnnnn	12,824	Government	Yes
@fffff	12,315	Citizen	No
@ggggg	10,564	Doctor	Yes
@hhhhh	10,310	Citizen	Unclear
@iiiii	10,172	Patient relative	Yes

**Table 3:** Top users (and their retweets) identified on Twitter/X.

Username	Retweets, n	Type	In favor of donation?
@bbbbb	16,392	Citizen	Unclear
@jjjjj	7674	Doctor	Yes
@hhhhh	7415	Citizen	Unclear
@aaaaa	6964	Citizen	Yes
@ccccc	6350	Doctor	No
@kkkkk	5471	Citizen	No
@ddddd	4491	Unknown	Yes
@nnnnn	3270	Government	Yes
@lllll	2448	Citizen	Yes
@mmmmm	2192	Doctor	Unclear

### Place Dimension: Countries, Regions, and Place Types

According to Table 4, places identified in YouTube videos were predominantly closed spaces (332/1317, 25.21%), hospitals (242/1317, 18.38%), and open spaces (205/1317, 15.57%). Significant differences were observed in hospitals (*F*_2.46,147.74_=–5.28, *P*=.005), medical offices (*F*_0.72,40.24_=–5.70, *P*=.003), closed places (*F*_3.44,155.78_=–7.02, *P*=.001), open places (*F*_2.31,136.81_=–5.37, *P*=.005), and unknown locations (*F*_3.25,78.30_=–13.18, *P*<.001) across ODIQ groups. Bonferroni tests revealed differences in means for various locations across video categories: hospitals differed between misinformation and no information (95% CI 0.04-0.30, *P*=.004); medical offices between information and misinformation (95% CI 0.02-0.15, *P*=.006); closed places between no information and information (95% CI 0.06-0.27, *P*=.001); open spaces between no information and information (95% CI 0.03-0.23, *P*=.006); and unknown locations between information and no information (95% CI 0.08-0.23, *P*<.001) and between information and misinformation (95% CI 0.03-0.22, *P*=.005).

The most commonly identified Japanese regions were unspecified (389/548, 70.9%), followed by Kanto (53/548, 9.7%). Significant differences were observed for Hokkaido (*F*_0.28,14.36_=–6.24, *P*=.002) and Kyushu and Okinawa (*F*_0.537,20.70_=–8.23, *P*<.001), with pairwise comparisons indicating variations between the means of Hokkaido in information and no information videos (95% CI 0.01-0.08, *P*=.003); and Kyushu and Okinawa in information and no information videos (95% CI 0.02-0.10, *P*=.001) and information and misinformation videos (95% CI 0.02-0.11, *P*=.004).

Significant differences were found among countries including Canada (*F*_0.45,25.39_=–5.72, *P*=.003), China (*F*_10.93,111.87_=–31.02, *P*<.001), India (*F*_0.27,15.32_=–5.76, *P*=.003), Israel (*F*_0.25,15.34_=–5.16, *P*=.006), the United Kingdom (*F*_0.96,60.56_=–5.07, *P*=.006), and the United States (*F*_4.17,131.99_=–10.04, *P*<.001). Bonferroni tests revealed significant differences between the means of Canada in misinformation and no information videos (95% CI 0.02-0.12, *P*=.003); China in misinformation and no information videos (95% CI 0.11-0.32, *P*<.001), misinformation and information videos (95% CI 0.25-0.48, *P*<.001), and no information and information videos (95% CI 0.06-0.24, *P*<.001); India in misinformation and no information videos (95% CI 0.02-0.10, *P*=.002); Israel in misinformation and no information videos (95% CI 0.01-0.09, *P*=.005); the United Kingdom in misinformation and information videos (95% CI 0.03-0.19, *P*=.005); and the United States in misinformation and no information videos (95% CI 0.03-0.26, *P*=.009) and misinformation and information videos (95% CI 0.11-0.35, *P*<.001). Furthermore, on Twitter/X, Japan was mentioned 7486 times, while China was mentioned 2640 times.

**Table 4:** Places, locations, and countries in YouTube videos based on Organ Donation Information Quality.

Place			Information (n=792), n		No information (n=1018), n		Misinformation (n=546), n		Total (n=2356), n
Hospital			74		107^a^		61^a^		242
Medical office			26^a^		15		3 ^a^		44
Educational institution			24		11		13		48
Home			48		77		31		156
Religious building			7		2		3		12
Other closed space			100^a^		163^a^		69		332
Nature			69		80		33		182
Open space			57^a^		103^a^		45		205
Unknown			57^a^		24^a^		15		96
**Location in Japan**									
	Hokkaido	12^a^		2		1		15	
	Tohoku	3		5		0		8	
	Kanto	18		26		9		53	
	Chubu	14		14		5		33	
	Kansai	6		8		3		17	
	Chugoku	6		4		1		11	
	Kyushu/Okinawa	17^a^		4		1		22	
	Unspecified	134		171		84		389	
**Country**									
	Canada	8		7^a^		12^a^		27	
	China	29^a^		76^a^		61^a^		166	
	India	6		2^a^		8^a^		16	
	Israel	5		3^a^		8^a^		16	
	United Kingdom	18^a^		28		23^a^		69	
	United States	54		86		57^a^		197	

^a^Statistically significant results.

### Activity Dimension in YouTube Videos and Tweets

The average ODIQ of 638 videos was 0.68, with significant differences in terms of brain death definition (*F*_2.46,147.74_=–5.28, *P*=.005), several death definitions (*F*_19.42,68.96_=–89.44, *P*<.001), organs (*F*_22.75,79.56_=–90.79, *P*<.001), donation card (*F*_23.21,67.16_=–109.73, *P*<.001), other donation statements (*F*_30.91,75.52_=–129.94, *P*<.001), and the donation process (*F*_1.26,23.67_=–16.94, *P*<.001).

Pairwise comparisons uncovered differences between the means of brain death definitions in information and no information videos (95% CI 0.10-0.20, *P*<.001); death definitions in information and no information videos (95% CI 0.32-0.46, *P*<.001), information and misinformation videos (95% CI 0.18-0.35, *P*<.001), and misinformation and no information videos (95% CI 0.03-0.21, *P*=.002); organs in information and no information videos (95% CI 0.35-0.50, *P*<.001), information and misinformation videos (95% CI 0.10-0.28, *P*<.001), and misinformation and no information videos (95% CI 0.14-0.32, *P*<.001); and donation card in information and no information videos (95% CI 0.35-0.49, *P*<.001) and information and misinformation videos (95% CI 0.24-0.42, *P*<.001).

We observed differences in the means of donation statements in information and no information videos (95% CI 0.42-0.56, *P*<.001), information and misinformation videos (95% CI 0.24-0.42, *P*<.001), and misinformation and no information videos (95% CI 0.07-0.25, *P*<.001); and donation process in information and no information videos (95% CI 0.06-0.14, *P*<.001) and information and misinformation videos (95% CI 0.02-0.13, *P*=.002). In information videos, ODIQ elements were presented more frequently than in misinformation videos. Specifically, the most common donation statements appeared in 115 out of 638 (18.0%) instances, followed by organs that can be donated in 99 out of 638 (15.5%) instances, the donation card in 98 out of 638 (15.4%) instances, and various definitions of death in 91 out of 638 (14.3%) instances. Further, we observed significant differences (family consent: *F*_8.50,53.03_=–50.91, *P*<.001; internet consent: *F*_1.68,22.34_=–23.87, *P*<.001) between types of donation statements, with family consent being the most frequent in information videos (60/638, 9.4%) and internet the least frequent (25/638, 3.9%).

Regarding video duration and views, we found significant differences (*H*_2_=37.38, *P*<.001 and *H*_2_=14.79, *P*<.001, respectively). Post hoc tests identified differences between the means of duration in no information and information videos (test statistic –69.46, *P*<.001) and no information and misinformation videos (test statistic 112.57, *P*<.001); and views in information and no information videos (test statistic 53.76, *P*=.003) and information and misinformation videos (test statistic 65.73, *P*=.004). Upon reviewing the 10 most viewed videos, we found that 6 were categorized as no information, 3 as information, and 1 as misinformation. These included 3 transplant short videos by a medical association, 2 news reports, 2 webtoons (digital comics), 1 reaction video from TikTok, 1 promotional campaign video, and 1 comedy dialog. Of the 2 webtoons, 1 contained information and the other contained misinformation. The comedy dialog included information on organ donation.

**Figure 3:**
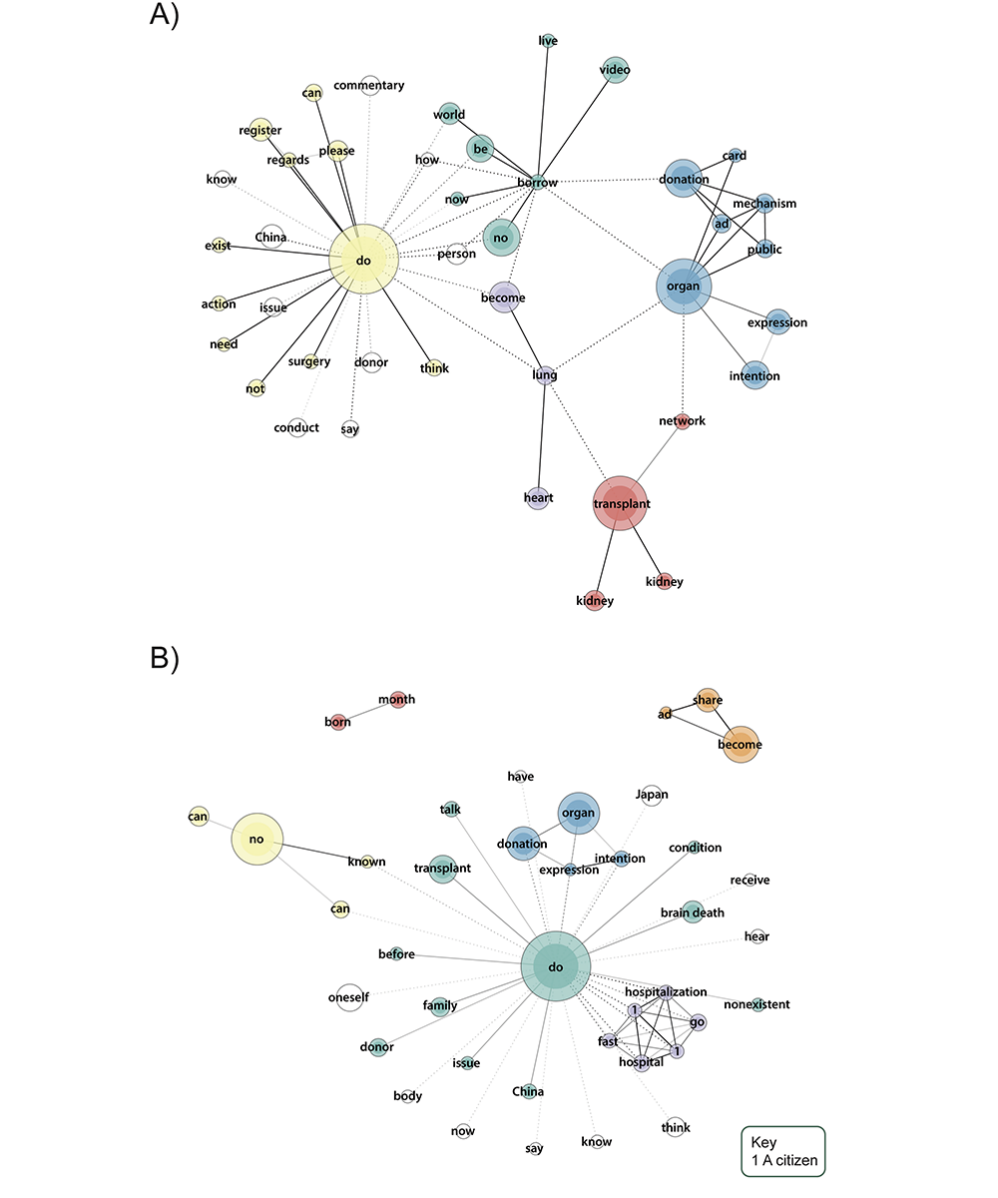
(A) Descriptions (word frequency 30-800, tie strength 0.96-1) and (B) comments (word frequency 120-4000, tie strength 0.7-1) of YouTube information videos.

**Figure 4:**
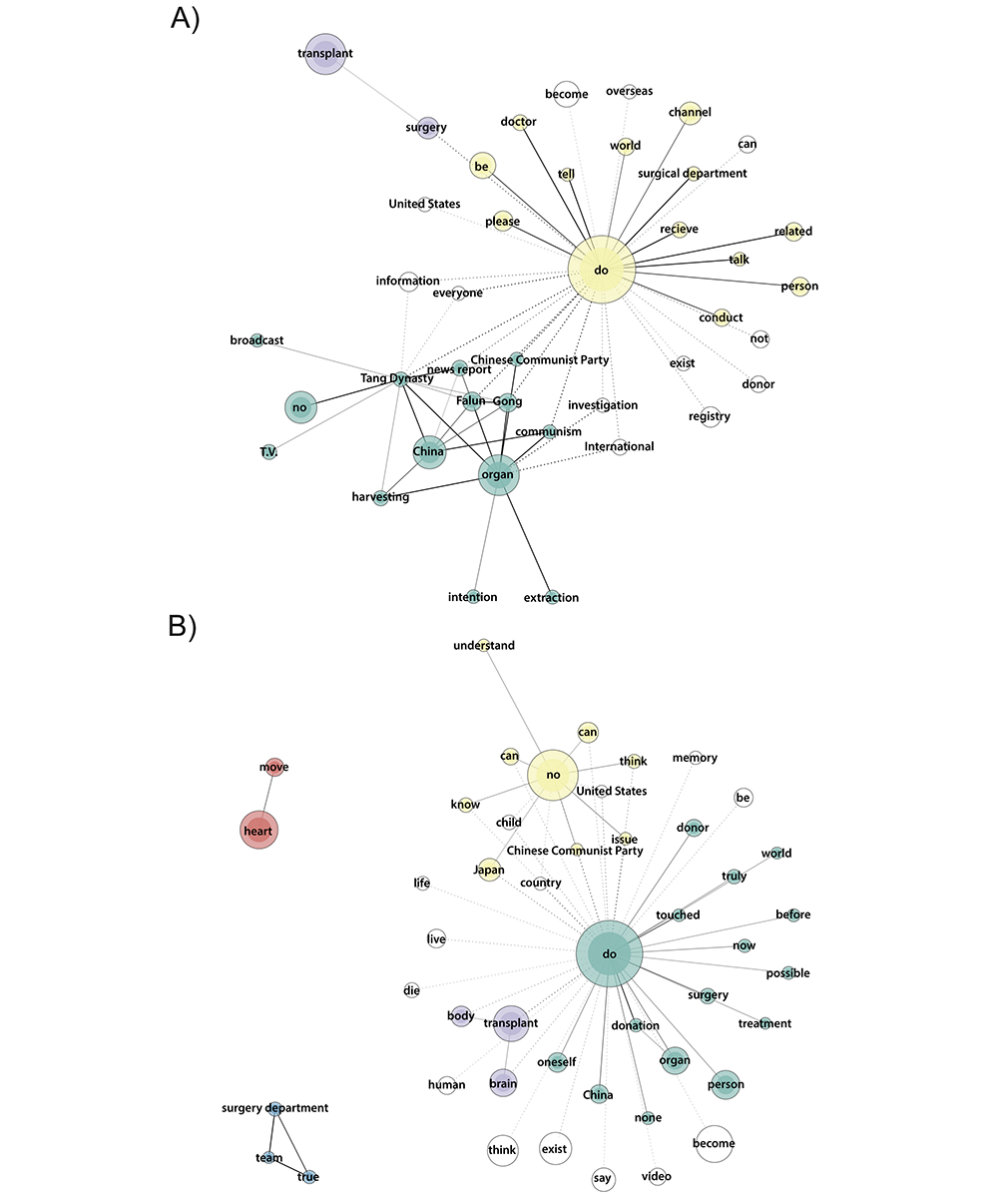
(A) Descriptions (word frequency 60-1500, tie strength 0.95-1) and (B) comments (word frequency 120-4000, tie strength 0.5-0.9) of YouTube no information videos.

**Figure 5:**
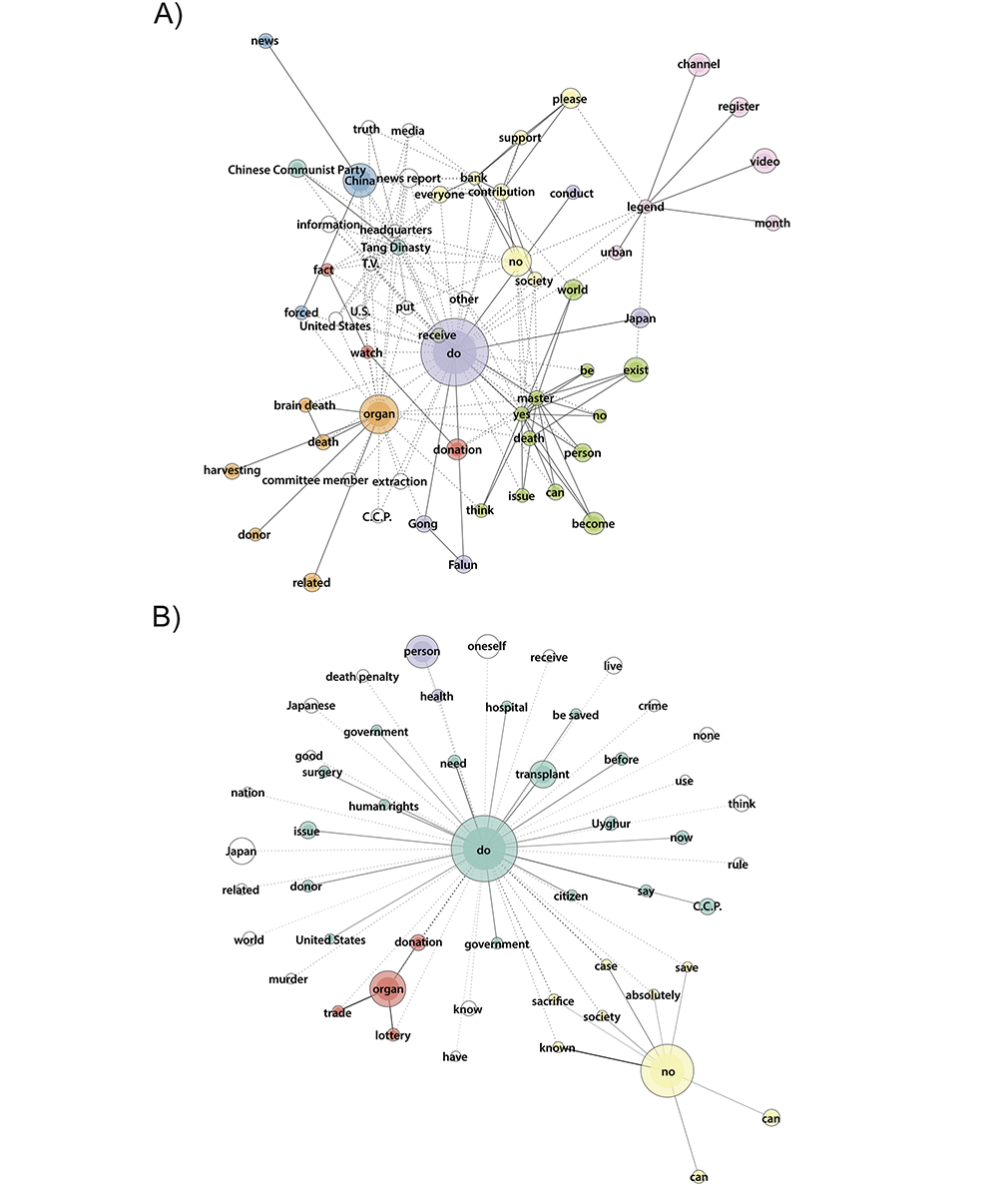
(A) Descriptions (word frequency 30-750, tie strength 0.6-1) and (B) comments (word frequency 150-6000, tie strength 0.65-0.85) of YouTube misinformation videos.

We analyzed the most common words used in video descriptions (Figures 3A-5A) and their corresponding comments (Figures 3B-5B) within ODIQ-based groups. Node size reflects word frequency, with thicker lines indicating stronger tie strengths. Nodes without color are not associated with any specific group. It is important to note that certain words with similar meanings in English can be expressed in multiple ways in Japanese. In information videos, a green cluster represents organ donation as borrowing life, uploaded by associations, while a yellow cluster of descriptions uploaded by various actors (mostly associations) encourages viewers to register as donors. In purple, a word group mentioned the lung and heart, connected to a red group representing the Organ Transplant Network and YouTubers uploading kidney transplantation videos. This content is also linked to a blue cluster featuring older organ donation promotion videos released by the Advertising Council Japan and uploaded by various accounts. Other commonly used words were “China” and “donor.”

When it comes to comments, the majority are supportive of organ donation. A green group includes terms such as “before,” “brain death,” “donor,” “China,” “family,” and “talk,” linked to a yellow group that holds a negative view of organ donation. A purple group contains recommendations to seek medical help. Another cluster in blue discusses the “expression” of “intention” to donate, while a cluster in orange shows a positive reaction toward the old promotion videos. Additionally, other frequently mentioned words were “Japan” and “oneself,” reflecting organ donation as a personal decision that requires individual consideration and awareness in Japan.

Regarding descriptions of no information videos (Figure 4), a green group includes news reports by Epoch Media Group YouTube channels (mentioning “Tang Dynasty”), an American organization with a branch office in Japan. These reports focus on accusations of organ trafficking involving some new religion practitioners in China. This group is linked to a yellow group consisting of interviews urging surgeons to refrain from international organ transplantation. Other notable terms were “United States” and “donor registry.”

Comments on these videos include a green cluster with the word “touched,” linked to a yellow cluster reacting to the organ trafficking videos. The red and blue clusters show positive reactions toward short videos released by a Japanese medical association about organ transplantation. Overall, comments regarding organ donation appear more proactive than negative.

Descriptions of misinformation videos were notably fragmented (Figure 5). A yellow group requested monetary donations to support Epoch Media Group channels, connected to a red group mentioning the term “fact,” and a blue group including the term “China.” In addition, a purple group linked some new religion practitioners to the term “Japan,” advocating to stop organ harvesting. Another cluster includes the terms “brain death,” “death,” “donor,” and “harvesting” in videos predominantly uploaded by Epoch Media Group channels and a few unknown actors.

By contrast, a lime green word group describes a video from a different new religion discussing resilience during the COVID-19 pandemic with a global perspective and a positive stance on organ donation. Additionally, a pink cluster describes videos uploaded on Japanese channels dedicated to urban legends, featuring stories of medical negligence during organ donation. Another term mentioned was “Chinese Communist Party.”

Regarding comments on misinformation videos, a green cluster indicates a positive reaction toward Epoch Media Group videos, particularly those addressing the persecution of Uyghur Muslim minorities in China as a human rights issue. A red word group associates organ donation with terms such as “lottery” and “trade.” Other words such as “crime,” “death penalty,” and “Japan” characterize organ donation in negative connotations.

**Figure 6:**
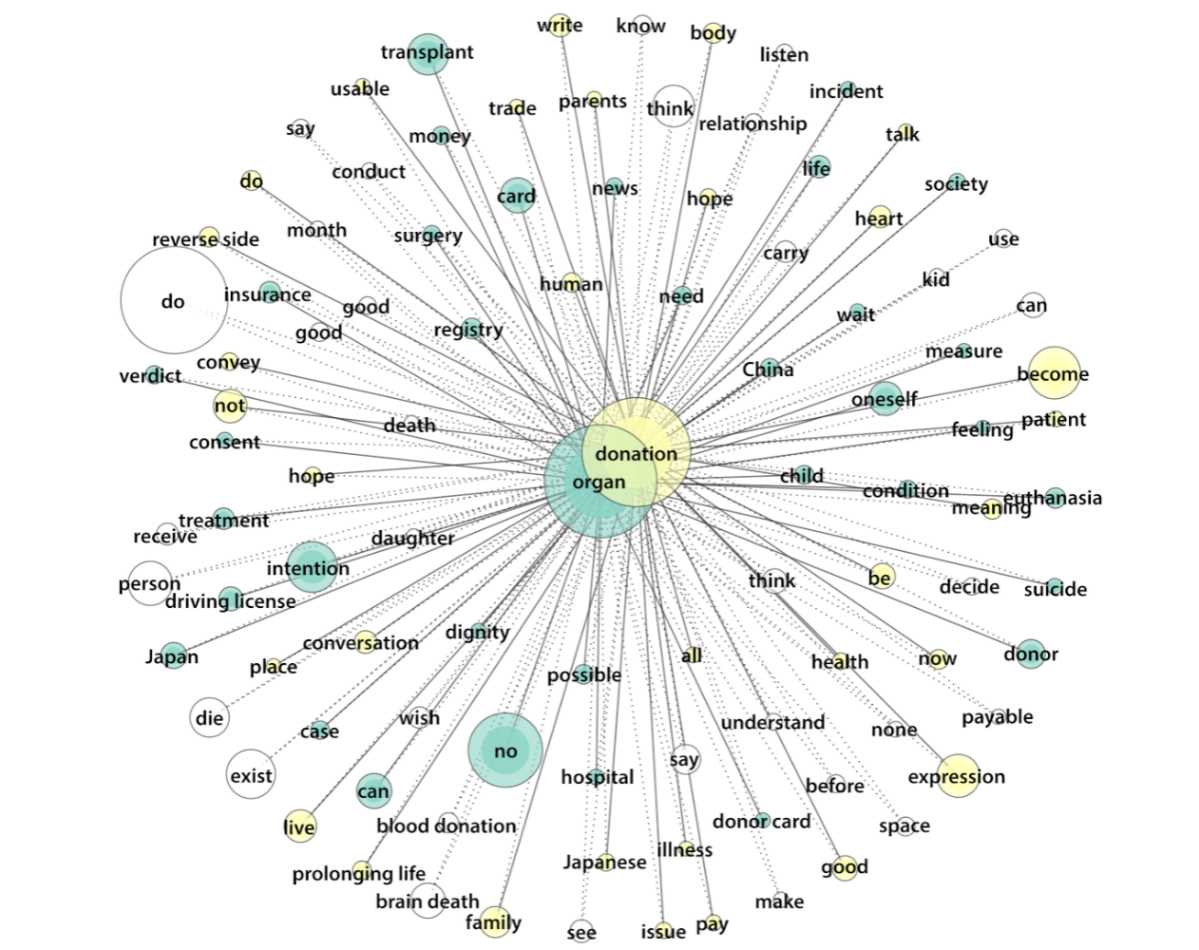
Top words in tweets (frequency 1750-125,000, tie strength 0.6-1).

In relation to Twitter/X, a prominent group of top words in green indicates statements about donation intentions through donation cards, driving licenses, and health insurance cards. This group includes mentions of “China,” “consent,” “dignity,” “donor,” “hospital,” “Japan,” “money,” and “society” (Figure 6). A yellow group indicates the consent of Japanese families and parents, with terms such as “human,” “pay,” “prolonging life,” “trade,” and “usable.” Other terms within this group express positivity toward donation (good) and include “blood donation” and “brain death.”

### Relations Dimension: Hyperlinks Beyond YouTube and Twitter/X

In the case of the 638 YouTube videos, 319 (50%) included social media hyperlinks, 317 (49.7%) linked to web pages, 46 (7.2%) included an email address, and 25 (3.9%) included a link to a blog. The ANOVA test revealed significant differences in social media links (*F*_4.75,154.50_=–9.74, *P*<.001) and email sharing (*F*_1.63,41.02_=–12.87, *P*<.001) across ODIQ groups. Post hoc tests indicated differences in the means of social media links between misinformation and information videos (95% CI 0.06-0.32, *P*=.002) and no information and information videos (95% CI 0.07-0.28, *P*<.001). Additionally, differences were found in email sharing between misinformation and no information videos (95% CI 0.03-0.17, *P*=.001) and misinformation and information videos (95% CI 0.07-0.21, *P*<.001). Most social media links were directed to Twitter/X and YouTube, with fewer links to Amazon and Facebook.

Regarding Twitter/X, the majority of links in the sample of 100,000 tweets were to news media reports, including 1061 tweets (1.06%) from Yahoo News, 784 tweets (0.78%) from the Japan Broadcasting Corporation (NHK), and 625 tweets (0.63%) from Asahi News, 1 of the 5 largest newspapers in Japan. The highest time peaks appear to coincide with a news report from Mainichi News (another large newspaper in Japan) covering uterus transplantation research by Keio University, and a YouTube video discussing body donation. Additionally, a link to a health insurance company’s content on end-of-life preparations (including statements on organ donation) was tweeted 1130 times (1.13%).

### Narrative Analysis: Organ and Body Donation on YouTube and Twitter/X

Table 5 compiles the narratives related to donation found in the 638 YouTube videos, comparing new narratives with those compiled in Multimedia Appendix 3. The ANOVA indicated significant differences in favor (*F*_6.90,114.45_=–19.14, *P*<.001), economic issues (*F*_2.13,50.59_=–13.39, *P*<.001), and organ trafficking (*F*_1.63,41.02_=–12.87, *P*<.001). Pairwise comparisons revealed differences in means of narratives related to favorability between no information and misinformation videos (95% CI 0.10-0.32, *P*<.001), and no information and information videos (95% CI 0.18-0.40, *P*<.001). Differences were also found in economic issues between misinformation and no information videos (95% CI 0.07-0.22, *P*<.001) and misinformation and information videos (95% CI 0.07-0.22, *P*<.001). Furthermore, disparities were observed in narratives about organ trafficking between misinformation and no information videos (95% CI 0.04-0.25, *P*=.004), misinformation and information videos (95% CI 0.25-0.47, *P*<.001), and no information and information videos (95% CI 0.13-0.31, *P*<.001).

Regarding religions mentioned in the 638 videos (Table 6), statistically significant differences were found in Christianity (*F*_0.29,17.19_=–5.50, *P*=.004), Islam (*F*_1.61,48.64_=–10.55, *P*<.001), and new religions (*F*_3.90,63.80_=–19.43, *P*<.001). Bonferroni tests indicated differences in the means of Christianity between misinformation and no information videos (95% CI 0.01-0.10, *P*=.007) and misinformation and information videos (95% CI 0.01-0.10, *P*=.008). Differences were also observed in Islam between misinformation and information videos (95% CI 0.06-0.21, *P*<.001). Additionally, disparities were found in new religions between misinformation and no information videos (95% CI 0.04-0.21, *P*=.001), misinformation and information videos (95% CI 0.13-0.30, *P*<.001), and no information and information videos (95% CI 0.02-0.16, *P*=.004).

**Table 5:** The main narratives tied to donation in YouTube videos based on Organ Donation Information Quality (N=638).

Narratives		Information (n=172), n (%)	No information (n=225), n (%)	Misinformation (n=42), n (%)	Total (n=322), n (%)
**Narratives in favor**					
	In favor	81 (12.7)	75 (11.8)	7 (1.1)^a^	163 (25.5)
	Connecting lives	9 (1.4)	8 (1.3)	1 (0.2)	18 (2.8)
	Ganbaru	6 (0.9)	10 (1.6)	3 (0.5)	19 (3.0)
	Green ribbon	14 (2.2)	15 (2.4)	0 (0)	29 (4.5)
	Helping others	13 (2.0)	4 (0.6)	1 (0.2)	18 (2.8)
**Narratives against**					
	Against	9 (1.4)	4 (0.6)	4 (0.6)	17 (2.7)
	Economic Issues	14 (2.2)	18 (2.8)	26 (4.1)^a^	58 (9.1)
	Organ trafficking	20 (3.1)^a^	85 (13.3)^a^	56 (8.8)^a^	161 (25.2)
**Unclear narratives**					
	Mechanistic view of life	6 (0.9)	6 (0.9)	5 (0.8)	17 (2.7)

^a^Statistically significant results.

**Table 6:** Religions in YouTube videos across Organ Donation Information Quality groups (N=638).

Religion	Information (n=27), n (%)	No information (n=75), n (%)	Misinformation (n=72), n (%)	Total (n=174), n (%)
Buddhism	6 (0.9)	4 (0.6)	7 (1.1)	17 (2.7)
Christianity	4 (0.6)	5 (0.8)	9 (1.4)^a^	18 (2.8)
Confucianism	0 (0)	1 (0.2)	0 (0)	1 (0.2)
Islam	7 (1.1)^a^	27 (4.2)	21 (3.3)^a^	55 (8.6)
Judaism	0 (0)	0 (0)	1 (0.2)	1 (0.2)
New religions	9 (1.4)^a^	36 (5.6)^a^	32 (5.0)^a^	77 (12.1)
Shinto	1 (0.2)	2 (0.3)	2 (0.3)	5 (0.8)

^a^Statistically significant results.

Regarding the 7 videos on donation for research, 5 were uploaded in 2022, 1 in 2021, and 1 in 2020. These videos were uploaded by 3 citizens (including 2 funerary staff), 2 educational actors, 2 media channels, 1 association, and 1 religious actor. A total of 5 videos featured citizens; 4 included doctors, donors, and associations; 3 involved other medical staff, educational actors, and media actors; and 1 each featured a medical student, a nurse, a recipient, a donor relative, another patient, a government functionary, and a religious actor. All videos depicted locations in Japan, with 3 showing the Kanto region. Among these, 3 of the videos were classified as information, 2 as no information, and 2 as misinformation. The information videos predominantly supported organ donation, providing explanations on ways to express consent and mentioning family consent. One video, shared by a funerary staff on Twitter/X, also detailed the history of body donation and the donation process.

Another video, uploaded by an inheritance consultation staff, discussed body donation as part of end-of-life care, detailed how to register for body donation, and highlighted organ donation as a gift of life according to the JOTN. This video was notable for its coverage of the organ extraction process, body preservation, and the absence of costs for organ donation by the donor. People in the comments reacted with curiosity. A video by an association featured several experts discussing international aspects of donation, presenting donation as a normal practice, the role of donor coordinators in research, and donation as part of a specific undergraduate course at a Spanish university. This video links donation activities with education and research, with the research results specifically related to heart and lung transplantation.

In no information videos, we observed negligence acting as a deterrent for body donation. One news report highlighted a case where a donor had specified their wish for their body to be used for medical education. However, after a few years, the university cremated the body without first contacting the family. A comment argued that the donation was for profit. Another video by funeral experts discusses a university that did not preserve the body correctly. The staff, being experts on death, were able to identify issues in the preservation process and body management, all while acknowledging the emotions of the bereaved relatives. Consequently, most comments sympathetically argued about human error and emphasized the necessity for unified guidelines and proper training in handling body donations.

Religion was mentioned in a misinformation video, and organ trafficking was highlighted in a news report, prompting reactions of fear, sadness, and anger in the comments. Another video resembled one of the informational videos about donor coordinators and normalizing organ donation, but it also included the for-profit economic narrative and racist content.

Regarding Twitter/X, Figure 6 illustrates that several prominent narratives emerged, including “death with dignity” related to preparing for a good death, a mechanistic perspective of life labeled as “usable,” and economic aspects referenced by terms such as “pay,” “payable,” and “trade.” The top commenters, based on retweets, supported blood donation, euthanasia, and organ donation, including donation from children, often expressed through donor cards and largely aligned with a mechanistic view of life. They also mentioned “Night Doctor,” a Japanese drama; “Never Let Me Go,” a novel by a Japanese author depicting a fictional future in England; and the anime “Angel Beats!.” Others discussed organ trafficking in China and emphasized the importance of respecting donors’ anonymity. Some expressed concern that organ donation might not be feasible for them due to parental disagreement, living alone, or having an illness.

Top commenters, based on likes, largely supported blood donation, euthanasia, and organ donation through written documents, alongside symbols such as the green ribbon, reflecting a mechanistic view of life. References were made to “Never Let Me Go” and “Angel Beats!.” Some users discussed body donation, economic concerns, and organ trafficking in China. Others expressed anxiety about their inability to donate due to family disagreements or personal health issues.

## Discussion

### Communication of Organ Donation Across Dimensions

In the context of YouTube, doctors and other medical staff, particularly donor coordinators, predominantly uploaded videos categorized as information. Associations and citizens contributed a few misinformation videos, while the media, religious actors, and educational entities primarily uploaded videos lacking substantive information. Doctors and citizens were less prevalent in misinformation videos. Other medical staff and associations were prominently featured in information videos. Patients, government officials, media figures, and religious actors were primarily represented in videos that did not contain medical information about organ donation. The public’s interest and active participation in disseminating organ donation–related information appear to surpass those of doctors, contrary to the findings of Ogawa et al [13]. However, the frequent mentions of heart transplantation and the presence of heart transplantation experts in the videos underscore the keen interest of this medical group.

Misinformation was less prevalent in hospitals, medical offices, and unknown locations, whereas videos lacking information predominantly featured closed and open spaces, which tended to be more urban than rural. Specifically, Hokkaido, Kyushu, and Okinawa are regions in Japan known for their cultural and ethnic diversity, suggesting a potential correlation between the dissemination of medical information on organ donation and regional diversity.

Canada, India, Israel, the United Kingdom, and the United States were prominently mentioned in misinformation videos, whereas China featured prominently in no information videos, followed closely by misinformation videos. Israel frequently appeared in contexts opposing organ trafficking, often in conjunction with accusations against China distributed by the Epoch Media Group. China was prominently featured on both YouTube and Twitter/X. A new religion was regularly discussed in no information and misinformation videos by these media outlets, employing the narrative of Gotai Manzoku in accusations related to organ trafficking, which was linked to Buddhism and Chinese culture. Furthermore, news reports utilized Christian imagery and highlighted Christian and Islamic communities in China as victims of organ trafficking.

The Epoch Media Group, founded by new religion adherents in New York in 1999 as a newspaper called The Epoch Times, has expanded into multiple media platforms in over 30 countries, including Japan. Initially focused on advocating for the religious rights of the outlawed new religion in China [38], it shifted toward far-right perspectives when reporting on Middle Eastern migration to the European Union in 2015 [39]. At its peak of advertising expenditure in the United States, the group’s videos collectively amassed around 3 billion views on Facebook, YouTube, and Twitter/X, ranking 11th among video creators across platforms and surpassing traditional news media [40]. Their connections with the far right could explain why India, Israel, and the United States are frequently cited in their content, given the escalation of far-right rhetoric and actions by governments in these countries in recent years.

In the realm of organ donation, some adherents of the new religion have accused the Chinese government of organ harvesting from prisoners since 2006 [41]. However, there is considerably less discussion about how members avoid modern medical treatments due to their belief that illness is caused by karma [42]. It was reported that their leader envisions science as an immoral religion because the Chinese government used science to discredit Qi Gong, the basis of exercises in some new religions and spiritual practices in China [41]. However, irregularities in organ acquisition by Chinese researchers were documented in a journal paper [43] and reported by the Japanese channels of the Epoch Media Group, indicating that this media outlet does not always disseminate misinformation. They do not share their religious beliefs with outsiders; instead, they seem to use talking points and imagery from other religions to oppose organ donation in Japan. Beyond social media environments, recent coalitions among some Japanese new religion practitioners have supported conservative politicians, influenced by practices learned from the American far right [44]. This could potentially impact legislation, opposing the interests of medical professionals, patients, and researchers.

### Information Quality on YouTube and Twitter/X

Donation statements commonly accepted were card-based systems relying on family consent, but not through the internet. Some individuals expressed distrust toward My Number, an ID registry intended for e-governance, which extended to the Japanese government. Historical records indicate opposition to both analog and digital systems, with laws protecting internet data proving ineffective, posing risks of discrimination resulting from data breaches [45].

Our data indicate proactive narratives of donation, such as “helping others,” among donor relatives and the public, aligning with findings previously observed among medical students [20]. Additionally, our findings highlight that the indirect communication style commonly favored by the Japanese supports organ donation indirectly, as evidenced by symbols such as the green ribbon. Old television commercials on YouTube related to organ donation may attract interest, partly due to nostalgia-driven trends in social media that focus on graphics, music, and multimedia. This nostalgia is associated with commercials originally airing between 1999 and 2007, as uploaded by the Advertising Council Japan and some citizens. This time frame roughly corresponds to the end of the economic bubble in the 1990s, before environmental disasters such as the Kumamoto Earthquake and the Fukushima nuclear reactor failure in 2011. Therefore, compared with the increasing economic inequalities and environmental damages in later years, this period seems to embody comfort, prosperity, and safety for the Japanese people [46].

While exploring innovative approaches to promote organ donation, we identified 3 YouTube videos on a channel managed by a woman kidney donor and a recipient. This platform discusses kidney illness, treatments, and interviews various stakeholders, aiming to normalize organ transplantation. Additionally, we encountered a video featuring a medical practitioner discussing the medical drama *Night Doctor,* in which one of the protagonists initially opposes organ donation by a family member. However, their perspective shifts after interacting with individuals involved in the donation process. Although emerging methods to communicate organ donation can be categorized as entertainment education, *Night Doctor* is particularly relevant as it takes advantage of a charismatic character with whom the viewer can identify or empathize and sends them on a journey where they emerge changed. This technique has been applied successfully to improve science literacy and public health using dramas across the world [47].

The ranking of views for different types of videos about organ donation is as follows: information videos were the least viewed (rank=283.19), followed by no information videos (rank=336.96) and misinformation videos (rank=348.93). Interestingly, among the top 7 most viewed videos on organ donation, 1 was an information video and 6 were no information videos. These popular videos were short and filmed vertically (likely with a smartphone), uploaded by a medical association. They featured a young doctor addressing technical questions (eg, how to preserve a heart for the transplant) and encouraged viewers to comment and ask further questions on the topic. Reactions to these videos were overwhelmingly positive and supportive of organ donation. The top no information video garnered 4,729,206 views, surpassing the top misinformation video, which had 1,753,898 views. This indicates that using viral formats can outperform the popularity of science misinformation, even when the latter is strategically tailored to gain priority through social media algorithms [48].

Indirect messages opposing organ donation on social media frequently highlight economic concerns and organ trafficking, contributing to our understanding of public sentiment (as discussed in Maeda et al [11]). These economic issues encompass suspicions of financial gain by those involved in organ donation, as well as wider disparities that could potentially ensure greater access to resources for the top 10% [49] or for men [50] in Japan. Therefore, we identified a need for more consistent and transparent information dissemination regarding the organ donation process and associated costs to the public. Additionally, tools should be developed to promote a more equitable distribution of organs, ensuring greater allocation to regions with higher prevalence of target diseases and enhancing access to organ donation services in rural areas [51]. Economic support for middle- and low-income patients, along with a gender-sensitive approach [52], should also be prioritized. Enhancing transparency in reporting organ transplantation data across gender, income levels, and geographical regions could facilitate the development of more inclusive and effective programs in this area.

Regarding body donation on YouTube, the acknowledgment of errors in the donation procedure and other medical mistakes, along with the adept management of emotions such as anger and grief by those featured in the videos, appears beneficial in stimulating proactive engagement in the comments section, even for videos lacking medical information. Furthermore, videos that provide more comprehensive ODIQ information tend to receive more positive feedback from the public.

On Twitter/X, discussions about organ donation were intertwined with topics such as death with dignity, considered as part of preparations for a peaceful passing by some businesses and citizens. This comes amidst a notable increase in the number of older individuals passing away in Japan and the ongoing professionalization of funeral services. Consequently, businesses focused on death-related services have thrived, with estimates suggesting a market value between 700 (equivalent to US $4.45 billion) and 891 billion yen (equivalent to US $5.66 billion) as of 2005 [53]. Despite the profit motive, it is anticipated that businesses involved in death-related services, along with their clients, will contribute to mainstreaming discussions about preparing for a dignified death in public discourse. This could potentially encompass considerations such as organ and body donation for research purposes.

### Toward a Strategy for New Medical Research Procedures

Given our findings, we propose the following aspects to implement new medical research procedures such as the RAP.

To shift focus from the dyad of donation-transplantation to a research-oriented scenario, the conceptualization of research must be considered first. Reciprocity-based messages have shown optimal effectiveness for organ donation [54]. According to collected social media data, proactive narratives about donation are associated with prosocial behavior (eg, connecting lives and aiding others, and to a lesser extent, mutual assistance). Therefore, the proposed research should incorporate a prosocial and reciprocal dimension aligned with *kyosei.* The data acquired for medical research should not be viewed as the property of researchers, but rather as borrowed data.

One challenge with research databases is the expectation that they should be eternal, which is largely impractical because of technological changes and limitations. Therefore, considering research data as borrowed emphasizes their temporal nature, with those overseeing them seen as stewards rather than owners. Both short- and long-term approaches to data stewardship and storage should be implemented. To prevent loss, mismanagement, and weaponization of research data, a diverse group of stewards should oversee them, receiving frequent training to ensure fair treatment and technological safety. Decisions regarding data allocation for specific research projects should also be made collectively.

In addition, clear documentation must be available for the obtained organs, tissues, and their related data, along with research outputs and communications with the public. This information, as well as the donation workflow, costs, and involved parties, should be easily accessible to donors and their families. An anonymized version should be made public, not only in the local language but also internationally. Additionally, interested patients and relatives can be trained to steward the data.

When managing donation programs for research, aiming for diversity and a horizontal organization among actors increases the likelihood of identifying issues before implementation. Specialized donation coordinators should be trained to understand the medical, managerial, and economic aspects of the programs to effectively communicate them to patients, relatives, the media, and the public. Researchers and other participants in the donation program ecosystem should be trained to handle emotions such as anger and grief in worst-case scenarios, such as the mishandling of research samples. They should also collaborate with insurance and funerary staff to manage these situations effectively.

In terms of potential donors, there has been an increasing number of cases of excessive body donations to Japanese universities, particularly in the Kansai region. This trend is attributed to the perception of body donation as a social service and the growing openness in discussing death, influenced by global environmental crises [55]. Our data indicate that individuals interested in organ donation, despite being diagnosed with cancer or other illnesses, often believe they are unable to donate. Such cases could be directed to the RAP.

When considering cultural aspects, those wishing to implement new medical research procedures should be mindful of narratives from new religions. If the target population for research programs lacks critical thinking skills and religious knowledge, enhancing religious literacy through multireligious educational programs is recommended. Many Japanese individuals struggle to differentiate between mainstream religious ideas and those of new religions [56]. Representatives from Buddhism, Shinto, Christianity, and Islam could collaborate in these initiatives.

In terms of communication and promotion, utilizing local and familiar contexts, akin to those in television advertisements by the Advertising Council Japan, may resonate with Japanese adults and youngsters. Researchers should also monitor popular cultural products that disseminate both accurate information and misinformation on medical science topics. Further, medical professionals should take a proactive approach to combat misinformation using comprehensive communication strategies. These strategies should encompass indirect support methods (eg, using symbols such as the green ribbon), viral formats (eg, short videos), and longer communication formats (eg, interviews involving multiple experts discussing scientific topics tailored for the public).

Lastly, promoting new medical research procedures could involve partnerships with living donors who possess communication skills or are willing to undergo training in medical topics. Additionally, collaborations with funerary and insurance experts could be beneficial. Some of these experts also participate in ENDEX, the end-of-life exhibition in Japan, which annually facilitates open discussions on death and preparations for a dignified passing.

Regarding the strengths and limitations of our analysis, the use of both quantitative and qualitative methods enabled us to explore various aspects such as communication actors, time frames, locations, activities, and hyperlinks. This approach provided a comprehensive understanding of content across 2 platforms that cater to different formats and audiences. However, the findings of this research specific to the Japanese context may not necessarily generalize to other countries and regions. Therefore, conducting further research on a global scale is recommended. Additionally, it is important to note that individual differences such as motivation were not fully explored in our study. As public opinion often varies without clear reasons stated, conducting additional focus groups and interviews with the public is recommended.

### Conclusions

We analyzed the communication of organ donation in Japanese social media using a multidimensional framework. Based on our findings, we have formulated recommendations for organ and tissue donation for medical research purposes, including in the RAP. Our conclusions are listed in Textbox 3.

**Study conclusions.**

1. Time dimension
  While YouTube videos reached their peak in 2021 and 2022, tweets were numerous from 2019 to 2022.
2. Individuality dimension
  Associations and unknown actors uploaded more information videos, while citizens and doctors appeared frequently in them.
  Media- and education-related actors uploaded videos, while citizens, doctors, and media actors appeared more frequently in videos without information.
  Citizens uploaded and appeared more frequently in misinformation videos.
  Citizens primarily tweeted average retweeted and liked tweets.
3. Place dimension
  The locations most identified in YouTube videos were closed spaces, hospitals, and open spaces.
  The majority of Japanese regions in the videos were unidentified, followed by the Kanto region.
  A high number of information videos depicted locations in Hokkaido, Kyushu, and Okinawa.
  The most mentioned countries on Twitter/X were Japan and China.
4. Activity dimension
  Organ donation was associated with themes of borrowing life and the donor registry, with predominantly positive comments.
  Organ donation was primarily associated with accusations of trafficking by American media in videos lacking medical information and containing misinformation.
  Comments on videos lacking information were mostly proactive toward donation, whereas comments on misinformation videos were negative.
  Few videos and tweets discussed organ donation processes and costs.
  YouTube videos about body donation were largely positive.
5. Relations dimension
  On YouTube, the majority of hyperlinks pointed to Twitter/X and YouTube, with fewer links directing to Amazon and Facebook.
  On Twitter/X, the majority of links pointed to news reports by Japanese media.
6. Recommendations
  Consider research data as borrowed data.
  Implement horizontal and diversified management of donation programs.
  Be mindful of scientific misinformation and trends in popular culture.

## Appendix

Multimedia Appendix 1 Actors classification.

Multimedia Appendix 2 Place classification.

Multimedia Appendix 3 Posthoc tests.

Multimedia Appendix 4 Narratives related to organ donation.

## Abbreviations

JOTN: Japan Organ Transplantation Network
NHK: Japan Broadcasting Corporation
ODIQ: Organ Donation Information Quality
RAP: Rapid Autopsy Program
