# Supplementary material for: Japanese Perception of Organ Donation and Implications for New Medical Technologies: Quantitative and Qualitative Social Media Analyses
Source: JMIR Form Res. 2024 Jul 19;8:e55797. doi: 10.2196/55797 (PMC11297371; doi:10.2196/55797)
Supplement: Multimedia Appendix 2 [file formative_v8i1e55797_app2.pdf]

Multimedia Appendix 2. Place classification

Place Type	Description
Hospital	Clinic, hospital, operation room, ambulance interior, etc.
Medical Office	Office in a medical context.
Educational Institution	Higher Education institution such as university, vocational training school, etc.
Home	Apartment, house, etc.
Religious Building	Building used for religious activities such as church, mosque, shrine, temple, etc.
Other closed space	Non-medical office, coffee shop, jail, museum, press room, etc.
Nature	Agricultural fields, forests, grasslands, lakes, parks, sea, sky, etc.
Open space	Parking lot, market, plaza, street, etc.
Unknown	Places that could not be classified in other categories.
