# Supplementary material for: Japanese Perception of Organ Donation and Implications for New Medical Technologies: Quantitative and Qualitative Social Media Analyses
Source: JMIR Form Res. 2024 Jul 19;8:e55797. doi: 10.2196/55797 (PMC11297371; doi:10.2196/55797)
Supplement: Multimedia Appendix 3 [file formative_v8i1e55797_app3.pdf]

Multimedia Appendix 3. Posthoc tests.

Bonferroni Multiple Comparisons - Uploaders and Speakers

Dependent Variable			Mean Difference (I-J)	Std. Error	Sig.	95% Confidence Interval Lower Bound	95% Confidence Interval Upper Bound
Uploader_Dr	Misinfo	No Info	-0.035	0.025	0.491	-0.10	0.03
		Info	-.098*	0.026	0.000	-0.16	-0.04
	No Info	Misinfo	0.035	0.025	0.491	-0.03	0.10
		Info	-.063*	0.021	0.007	-0.11	-0.01
	Info	Misinfo	**.098***	**0.026**	**0.000**	**0.04**	**0.16**
		No Info	**.063***	**0.021**	**0.007**	**0.01**	**0.11**
Uploader_Other Medical Staff	Misinfo	No Info	0.001	0.016	1.000	-0.04	0.04
		Info	-.043	0.017	0.030	-0.08	0.00
	No Info	Misinfo	-0.001	0.016	1.000	-0.04	0.04
		Info	-.044*	0.013	0.003	-0.08	-0.01
	Info	Misinfo	.043	0.017	0.030	0.00	0.08
		No Info	**.044***	**0.013**	**0.003**	**0.01**	**0.08**
Uploader_Association	Misinfo	No Info	-.089	0.036	0.044	-0.18	0.00
		Info	**-.122***	**0.037**	**0.003**	**-0.21**	**-0.03**
	No Info	Misinfo	.089	0.036	0.044	0.00	0.18
		Info	-0.034	0.030	0.782	-0.11	0.04
	Info	Misinfo	.122*	0.037	0.003	0.03	0.21
		No Info	0.034	0.030	0.782	-0.04	0.11
Uploader_Citizen	Misinfo	No Info	**.177***	**0.048**	**0.001**	**0.06**	**0.29**
		Info	**.181***	**0.050**	**0.001**	**0.06**	**0.30**
	No Info	Misinfo	-.177*	0.048	0.001	-0.29	-0.06
		Info	0.004	0.040	1.000	-0.09	0.10
	Info	Misinfo	-.181*	0.050	0.001	-0.30	-0.06
		No Info	-0.004	0.040	1.000	-0.10	0.09
Uploader_Media	Misinfo	No Info	-0.055	0.047	0.743	-0.17	0.06
		Info	0.101	0.048	0.111	-0.01	0.22
	No Info	Misinfo	0.055	0.047	0.743	-0.06	0.17
		Info	**.156***	**0.039**	**0.000**	**0.06**	**0.25**
	Info	Misinfo	-0.101	0.048	0.111	-0.22	0.01
		No Info	-.156*	0.039	0.000	-0.25	-0.06
Uploader_Religion	Misinfo	No Info	**.115***	**0.036**	**0.004**	**0.03**	**0.20**
		Info	**.180***	**0.037**	**0.000**	**0.09**	**0.27**

	No Info	Misinfo	-.115*	0.036	0.004	-0.20	-0.03
		Info	0.065	0.029	0.083	-0.01	0.14
	Info	Misinfo	-.180*	0.037	0.000	-0.27	-0.09
		No Info	-0.065	0.029	0.083	-0.14	0.01
Uploader_Edu	Misinfo	No Info	0.021	0.021	0.943	-0.03	0.07
		Info	-0.049	0.022	0.073	-0.10	0.00
	No Info	Misinfo	-0.021	0.021	0.943	-0.07	0.03
		Info	-.070*	0.017	0.000	-0.11	-0.03
	Info	Misinfo	0.049	0.022	0.073	0.00	0.10
		No Info	**.070***	**0.017**	**0.000**	**0.03**	**0.11**
Speaker_Dr	Misinfo	No Info	**.172***	**0.053**	**0.004**	**0.04**	**0.30**
		Info	0.077	0.054	0.472	-0.05	0.21
	No Info	Misinfo	-.172*	0.053	0.004	-0.30	-0.04
		Info	-0.095	0.044	0.088	-0.20	0.01
	Info	Misinfo	-0.077	0.054	0.472	-0.21	0.05
		No Info	0.095	0.044	0.088	-0.01	0.20
Speaker_Other Medical Staff	Misinfo	No Info	0.014	0.029	1.000	-0.06	0.08
		Info	-0.064	0.030	0.105	-0.14	0.01
	No Info	Misinfo	-0.014	0.029	1.000	-0.08	0.06
		Info	**-.077***	**0.024**	**0.004**	**-0.14**	**-0.02**
	Info	Misinfo	0.064	0.030	0.105	-0.01	0.14
		No Info	.077*	0.024	0.004	0.02	0.14
Speaker_Other Patient	Misinfo	No Info	-.136*	0.038	0.001	-0.23	-0.04
		Info	-0.031	0.039	1.000	-0.12	0.06
	No Info	Misinfo	**.136***	**0.038**	**0.001**	**0.04**	**0.23**
		Info	**.105***	**0.031**	**0.003**	**0.03**	**0.18**
	Info	Misinfo	0.031	0.039	1.000	-0.06	0.12
		No Info	-.105*	0.031	0.003	-0.18	-0.03
Speaker_Association	Misinfo	No Info	0.045	0.048	1.000	-0.07	0.16
		Info	-0.102	0.049	0.118	-0.22	0.02
	No Info	Misinfo	-0.045	0.048	1.000	-0.16	0.07
		Info	-.147*	0.040	0.001	-0.24	-0.05
	Info	Misinfo	0.102	0.049	0.118	-0.02	0.22
		No Info	**.147***	**0.040**	**0.001**	**0.05**	**0.24**
Speaker_Citizen	Misinfo	No Info	**.158***	**0.053**	**0.010**	**0.03**	**0.29**
		Info	**.178***	**0.055**	**0.004**	**0.05**	**0.31**
	No Info	Misinfo	-.158*	0.053	0.010	-0.29	-0.03
		Info	0.021	0.044	1.000	-0.08	0.13
	Info	Misinfo	-.178*	0.055	0.004	-0.31	-0.05
		No Info	-0.021	0.044	1.000	-0.13	0.08

Speaker_Government	Misinfo	No Info	**.219***	**0.047**	**0.000**	**0.11**	**0.33**
		Info	**.235***	**0.048**	**0.000**	**0.12**	**0.35**
	No Info	Misinfo	-.219*	0.047	0.000	-0.33	-0.11
		Info	0.016	0.039	1.000	-0.08	0.11
	Info	Misinfo	-.235*	0.048	0.000	-0.35	-0.12
		No Info	-0.016	0.039	1.000	-0.11	0.08
Speaker_Media	Misinfo	No Info	0.052	0.050	0.892	-0.07	0.17
		Info	.178*	0.051	0.002	0.06	0.30
	No Info	Misinfo	-0.052	0.050	0.892	-0.17	0.07
		Info	.126*	0.041	0.006	0.03	0.22
	Info	Misinfo	**-.178***	**0.051**	**0.002**	**-0.30**	**-0.06**
		No Info	**-.126***	**0.041**	**0.006**	**-0.22**	**-0.03**
Speaker_Religion	Misinfo	No Info	**.171***	**0.041**	**0.000**	**0.07**	**0.27**
		Info	**.221***	**0.042**	**0.000**	**0.12**	**0.32**
	No Info	Misinfo	-.171*	0.041	0.000	-0.27	-0.07
		Info	0.051	0.033	0.391	-0.03	0.13
	Info	Misinfo	-.221*	0.042	0.000	-0.32	-0.12
		No Info	-0.051	0.033	0.391	-0.13	0.03

*. The mean difference is significant at the 0.01 level.

Bonferroni Multiple Comparisons - Place

Dependent Variable			Mean Difference (I-J)	Std. Error	Sig.	95% Confidence Interval	
						Lower Bound	Upper Bound
Hospital	Misinfo	No Info	0.103	0.052	0.143	-0.02	0.23
		Info	**.173***	**0.053**	**0.004**	**0.04**	**0.30**
	No Info	Misinfo	-0.103	0.052	0.143	-0.23	0.02
		Info	0.070	0.043	0.306	-0.03	0.17
	Info	Misinfo	-.173*	0.053	0.004	-0.30	-0.04
		No Info	-0.070	0.043	0.306	-0.17	0.03
Medical office	Misinfo	No Info	-0.030	0.027	0.809	-0.10	0.04
		Info	-.087*	0.028	0.006	-0.15	-0.02
	No Info	Misinfo	0.030	0.027	0.809	-0.04	0.10
		Info	-.057	0.022	0.034	-0.11	0.00
	Info	Misinfo	**.087***	**0.028**	**0.006**	**0.02**	**0.15**
		No Info	.057	0.022	0.034	0.00	0.11
Other Closed	Misinfo	No Info	-0.034	0.053	1.000	-0.16	0.09
		Info	0.126	0.055	0.064	-0.01	0.26

	No Info	Misinfo	0.034	0.053	1.000	-0.09	0.16
		Info	**.161***	**0.044**	**0.001**	**0.06**	**0.27**
	Info	Misinfo	-0.126	0.055	0.064	-0.26	0.01
		No Info	-.161*	0.044	0.001	-0.27	-0.06
Open	Misinfo	No Info	-0.011	0.050	1.000	-0.13	0.11
		Info	0.117	0.051	0.068	-0.01	0.24
	No Info	Misinfo	0.011	0.050	1.000	-0.11	0.13
		Info	**.128***	**0.041**	**0.006**	**0.03**	**0.23**
	Info	Misinfo	-0.117	0.051	0.068	-0.24	0.01
		No Info	-.128*	0.041	0.006	-0.23	-0.03
Unknown	Misinfo	No Info	0.034	0.038	1.000	-0.06	0.12
		Info	-.123*	0.039	0.005	-0.22	-0.03
	No Info	Misinfo	-0.034	0.038	1.000	-0.12	0.06
		Info	-.156*	0.031	0.000	-0.23	-0.08
	Info	Misinfo	**.123***	**0.039**	**0.005**	**0.03**	**0.22**
		No Info	**.156***	**0.031**	**0.000**	**0.08**	**0.23**

*. The mean difference is significant at the 0.01 level.

Bonferroni Multiple Comparisons – Location in Japan

Dependent Variable			Mean Difference (I-J)	Std. Error	Sig.	95% Confidence Interval	
						Lower Bound	Upper Bound
Hokkaido	Misinfo	No Info	0.001	0.016	1.000	-0.04	0.04
		Info	-.043	0.017	0.030	-0.08	0.00
	No Info	Misinfo	-0.001	0.016	1.000	-0.04	0.04
		Info	-.044*	0.013	0.003	-0.08	-0.01
	Info	Misinfo	.043	0.017	0.030	0.00	0.08
		No Info	**.044***	**0.013**	**0.003**	**0.01**	**0.08**
Kyuushuu and Okinawa	Misinfo	No Info	-0.006	0.019	1.000	-0.05	0.04
		Info	-.064*	0.020	0.004	-0.11	-0.02
	No Info	Misinfo	0.006	0.019	1.000	-0.04	0.05
		Info	-.058*	0.016	0.001	-0.10	-0.02
	Info	Misinfo	**.064***	**0.020**	**0.004**	**0.02**	**0.11**
		No Info	**.058***	**0.016**	**0.001**	**0.02**	**0.10**

*. The mean difference is significant at the 0.01 level.

Bonferroni Multiple Comparisons - Countries

Dependent Variable			Mean Difference (I-J)	Std. Error	Sig.	95% Confidence Interval	
						Lower Bound	Upper Bound
Canada	Misinfo	No Info	**.071***	**0.022**	**0.003**	**0.02**	**0.12**
		Info	.062	0.022	0.016	0.01	0.12
	No Info	Misinfo	-.071*	0.022	0.003	-0.12	-0.02
		Info	-0.009	0.018	1.000	-0.05	0.03
	Info	No Info	-.062	0.022	0.016	-0.12	-0.01
		No Info	0.009	0.018	1.000	-0.03	0.05
China	Misinfo	No Info	**.215***	**0.045**	**0.000**	**0.11**	**0.32**
		Info	**.365***	**0.046**	**0.000**	**0.25**	**0.48**
	No Info	Misinfo	-.215*	0.045	0.000	-0.32	-0.11
		Info	**.150***	**0.037**	**0.000**	**0.06**	**0.24**
	Info	Misinfo	-.365*	0.046	0.000	-0.48	-0.25
		No Info	-.150*	0.037	0.000	-0.24	-0.06
India	Misinfo	No Info	**.057***	**0.017**	**0.002**	**0.02**	**0.10**
		Info	0.038	0.017	0.077	0.00	0.08
	No Info	Misinfo	-.057*	0.017	0.002	-0.10	-0.02
		Info	-0.018	0.014	0.550	-0.05	0.01
	Info	Misinfo	-0.038	0.017	0.077	-0.08	0.00
		No Info	0.018	0.014	0.550	-0.01	0.05
Israel	Misinfo	No Info	**.053***	**0.017**	**0.005**	**0.01**	**0.09**
		Info	.043	0.017	0.040	0.00	0.08
	No Info	Misinfo	-.053*	0.017	0.005	-0.09	-0.01
		Info	-0.010	0.014	1.000	-0.04	0.02
	Info	Misinfo	-.043	0.017	0.040	-0.08	0.00
		No Info	0.010	0.014	1.000	-0.02	0.04
UK	Misinfo	No Info	.083*	0.033	0.038	0.00	0.16
		Info	**.107***	**0.034**	**0.005**	**0.03**	**0.19**
	No Info	Misinfo	-.083*	0.033	0.038	-0.16	0.00
		Info	0.024	0.027	1.000	-0.04	0.09
	Info	Misinfo	-.107*	0.034	0.005	-0.19	-0.03
		No Info	-0.024	0.027	1.000	-0.09	0.04
US	Misinfo	No Info	**.147***	**0.049**	**0.009**	**0.03**	**0.26**
		Info	**.226***	**0.050**	**0.000**	**0.11**	**0.35**
	No Info	Misinfo	-.147*	0.049	0.009	-0.26	-0.03
		Info	0.080	0.040	0.148	-0.02	0.18

Info	Misinfo	-.226*	0.050	0.000	-0.35	-0.11
	No Info	-0.080	0.040	0.148	-0.18	0.02

*. The mean difference is significant at the 0.01 level.

Bonferroni Multiple Comparisons – Organ Donation Information Quality (ODIQ)

Dependent Variable			Mean Difference (I-J)	Std. Error	Sig.	95% Confidence Interval Lower Bound	Upper Bound
ODIQ1_Definition	Misinfo	No Info	.072	0.026	0.020	0.01	0.14
		Info	-.077	0.027	0.014	-0.14	-0.01
	No Info	Misinfo	-.072	0.026	0.020	-0.14	-0.01
		Info	-.149*	0.022	0.000	-0.20	-0.10
	Info	Misinfo	.077	0.027	0.014	0.01	0.14
		No Info	**.149***	**0.022**	**0.000**	**0.10**	**0.20**
ODIQ2_Several Definitions	Misinfo	No Info	**.120***	**0.035**	**0.002**	**0.03**	**0.21**
		Info	-.267*	0.036	0.000	-0.35	-0.18
	No Info	Misinfo	-.120*	0.035	0.002	-0.21	-0.03
		Info	-.387*	0.029	0.000	-0.46	-0.32
	Info	Misinfo	**.267***	**0.036**	**0.000**	**0.18**	**0.35**
		No Info	**.387***	**0.029**	**0.000**	**0.32**	**0.46**
ODIQ3_Organs	Misinfo	No Info	**.232***	**0.038**	**0.000**	**0.14**	**0.32**
		Info	-.189*	0.039	0.000	-0.28	-0.10
	No Info	Misinfo	-.232*	0.038	0.000	-0.32	-0.14
		Info	-.421*	0.031	0.000	-0.50	-0.35
	Info	Misinfo	**.189***	**0.039**	**0.000**	**0.10**	**0.28**
		No Info	**.421***	**0.031**	**0.000**	**0.35**	**0.50**
ODIQ4_Organ Donation Card	Misinfo	No Info	.088	0.035	0.037	0.00	0.17
		Info	-.329*	0.036	0.000	-0.42	-0.24
	No Info	Misinfo	-.088	0.035	0.037	-0.17	0.00
		Info	-.417*	0.029	0.000	-0.49	-0.35
	Info	Misinfo	**.329***	**0.036**	**0.000**	**0.24**	**0.42**
		No Info	**.417***	**0.029**	**0.000**	**0.35**	**0.49**
ODIQ5_Other Donation Statements	Misinfo	No Info	**.160***	**0.037**	**0.000**	**0.07**	**0.25**
		Info	-.329*	0.038	0.000	-0.42	-0.24
	No Info	Misinfo	-.160*	0.037	0.000	-0.25	-0.07

		Info	-.489*	0.031	0.000	-0.56	-0.42
	Info	Misinfo	**.329***	**0.038**	**0.000**	**0.24**	**0.42**
		No Info	**.489***	**0.031**	**0.000**	**0.42**	**0.56**
ODIQ6_Donation Process	Misinfo	No Info	0.024	0.021	0.747	-0.03	0.07
		Info	-.074*	0.021	0.002	-0.13	-0.02
	No Info	Misinfo	-0.024	0.021	0.747	-0.07	0.03
		Info	-.098*	0.017	0.000	-0.14	-0.06
	Info	Misinfo	**.074***	**0.021**	**0.002**	**0.02**	**0.13**
		No Info	**.098***	**0.017**	**0.000**	**0.06**	**0.14**
ODIQ Total	Misinfo	No Info	-.280	0.098	0.013	-0.51	-0.05
		Info	-2.263*	0.100	0.000	-2.50	-2.02
	No Info	Misinfo	.280	0.098	0.013	0.05	0.51
		Info	-1.983*	0.080	0.000	-2.18	-1.79
	Info	Misinfo	**2.263***	**0.100**	**0.000**	**2.02**	**2.50**
		No Info	**1.983***	**0.080**	**0.000**	**1.79**	**2.18**
Death definition Type_Law	Misinfo	No Info	.088*	0.031	0.015	0.01	0.16
		Info	-.155*	0.032	0.000	-0.23	-0.08
	No Info	Misinfo	-.088*	0.031	0.015	-0.16	-0.01
		Info	-.243*	0.026	0.000	-0.30	-0.18
	Info	Misinfo	**.155***	**0.032**	**0.000**	**0.08**	**0.23**
		No Info	**.243***	**0.026**	**0.000**	**0.18**	**0.30**
Death definition Type_Heart Death	Misinfo	No Info	0.056	0.028	0.148	-0.01	0.12
		Info	-.144*	0.029	0.000	-0.21	-0.07
	No Info	Misinfo	-0.056	0.028	0.148	-0.12	0.01
		Info	-.200*	0.023	0.000	-0.26	-0.14
	Info	Misinfo	**.144***	**0.029**	**0.000**	**0.07**	**0.21**
		No Info	**.200***	**0.023**	**0.000**	**0.14**	**0.26**
Donation Statement Type_Health Insurance Card	Misinfo	No Info	0.056	0.028	0.138	-0.01	0.12
		Info	-.135*	0.029	0.000	-0.20	-0.07
	No Info	Misinfo	-0.056	0.028	0.138	-0.12	0.01
		Info	-.191*	0.023	0.000	-0.25	-0.14
	Info	Misinfo	**.135***	**0.029**	**0.000**	**0.07**	**0.20**
		No Info	**.191***	**0.023**	**0.000**	**0.14**	**0.25**
Donation Statement Type_Driving License	Misinfo	No Info	0.064	0.028	0.069	0.00	0.13
		Info	-.123*	0.029	0.000	-0.19	-0.05
	No Info	Misinfo	-0.064	0.028	0.069	-0.13	0.00
		Info	-.187*	0.023	0.000	-0.24	-0.13

	Info	Misinfo	**.123***	**0.029**	**0.000**	**0.05**	**0.19**
		No Info	**.187***	**0.023**	**0.000**	**0.13**	**0.24**
Donation Statement Type_My Number Card	Misinfo	No Info	0.008	0.023	1.000	-0.05	0.06
		Info	-.124*	0.023	0.000	-0.18	-0.07
	No Info	Misinfo	-0.008	0.023	1.000	-0.06	0.05
		Info	-.132*	0.019	0.000	-0.18	-0.09
	Info	Misinfo	**.124***	**0.023**	**0.000**	**0.07**	**0.18**
		No Info	**.132***	**0.019**	**0.000**	**0.09**	**0.18**
Donation Statement Type_Internet	Misinfo	No Info	0.000	0.020	1.000	-0.05	0.05
		Info	-.106*	0.021	0.000	-0.16	-0.06
	No Info	Misinfo	0.000	0.020	1.000	-0.05	0.05
		Info	-.106*	0.017	0.000	-0.15	-0.07
	Info	Misinfo	**.106***	**0.021**	**0.000**	**0.06**	**0.16**
		No Info	**.106***	**0.017**	**0.000**	**0.07**	**0.15**
Donation Statement Type_Family Consent	Misinfo	No Info	0.072	0.031	0.063	0.00	0.15
		Info	-.183*	0.032	0.000	-0.26	-0.11
	No Info	Misinfo	-0.072	0.031	0.063	-0.15	0.00
		Info	-.255*	0.026	0.000	-0.32	-0.19
	Info	Misinfo	**.183***	**0.032**	**0.000**	**0.11**	**0.26**
		No Info	**.255***	**0.026**	**0.000**	**0.19**	**0.32**

*. The mean difference is significant at the 0.01 level.

Pairwise Comparisons - Video duration (seconds)

Sample 1-Sample 2	Test Statistic	Std. Error	Std. Test Statistic	Sig.	Adj. Sig.[a]
No Info-Info	**-69.463***	**16.333**	**-4.253**	**0.000**	**0.000**
No Info-Misinfo	**112.574***	**19.849**	**5.671**	**0.000**	**0.000**
Info-Misinfo	43.111	20.405	2.113	0.035	0.104

Pairwise Comparisons - Video views

Sample 1-Sample 2	Test Statistic	Std. Error	Std. Test Statistic	Sig.	Adj. Sig.[a]
Info-No Info	**53.765***	**16.333**	**3.292**	**0.001**	**0.003**
Info-Misinfo	**65.734***	**20.405**	**3.222**	**0.001**	**0.004**
No Info-Misinfo	11.969	19.849	0.603	0.546	1.000

*. Ranks differences are significant at the 0.01 level.

Bonferroni Multiple Comparisons - Hyperlinks

Dependent Variable	Std. Error	Sig.	95% Confidence Interval

			Mean Difference (I-J)			Lower Bound	Upper Bound
Email	Misinfo	No Info	**.100***	**0.027**	**0.001**	**0.03**	**0.17**
		Info	**.142***	**0.028**	**0.000**	**0.07**	**0.21**
	Misinfo	No Info	-.100*	0.027	0.001	-0.17	-0.03
		Info	0.043	0.023	0.173	-0.01	0.10
	Info	Misinfo	-.142*	0.028	0.000	-0.21	-0.07
		No Info	-0.043	0.023	0.173	-0.10	0.01
SNS	Misinfo	No Info	0.015	0.053	1.000	-0.11	0.14
		Info	**.189***	**0.055**	**0.002**	**0.06**	**0.32**
	No Info	Misinfo	-0.015	0.053	1.000	-0.14	0.11
		Info	**.174***	**0.044**	**0.000**	**0.07**	**0.28**
	Info	Misinfo	-.189*	0.055	0.002	-0.32	-0.06
		No Info	-.174*	0.044	0.000	-0.28	-0.07

*. The mean difference is significant at the 0.01 level.

Bonferroni Multiple Comparisons - Narratives

Dependent Variable			Mean Difference (I-J)	Std. Error	Sig.	95% Confidence Interval	
						Lower Bound	Upper Bound
In favor	Misinfo	No Info	-.214*	0.046	0.000	-0.32	-0.10
		Info	-.289*	0.047	0.000	-0.40	-0.18
	No Info	Misinfo	**.214***	**0.046**	**0.000**	**0.10**	**0.32**
		Info	-0.075	0.038	0.141	-0.17	0.02
	Info	Misinfo	**.289***	**0.047**	**0.000**	**0.18**	**0.40**
		No Info	0.075	0.038	0.141	-0.02	0.17
Economic	Misinfo	No Info	**.143***	**0.030**	**0.000**	**0.07**	**0.22**
		Info	**.148***	**0.031**	**0.000**	**0.07**	**0.22**
	No Info	Misinfo	-.143*	0.030	0.000	-0.22	-0.07
		Info	0.005	0.025	1.000	-0.05	0.07
	Info	Misinfo	-.148*	0.031	0.000	-0.22	-0.07
		No Info	-0.005	0.025	1.000	-0.07	0.05
Organ Trafficking	Misinfo	No Info	**.142***	**0.044**	**0.004**	**0.04**	**0.25**
		Info	**.363***	**0.046**	**0.000**	**0.25**	**0.47**
	No Info	Misinfo	-.142*	0.044	0.004	-0.25	-0.04
		Info	**.221***	**0.037**	**0.000**	**0.13**	**0.31**

1Info	Misinfo	-.363*	0.046	0.000	-0.47	-0.25
	No Info	-.221*	0.037	0.000	-0.31	-0.13

*. The mean difference is significant at the 0.01 level.

Bonferroni Multiple Comparisons - Religion

Dependent Variable			Mean Difference (I-J)	Std. Error	Sig.	95% Confidence Interval Lower Bound	Upper Bound
Christianity	Misinfo	No Info	**.054***	**0.018**	**0.007**	**0.01**	**0.10**
		Info	**.055***	**0.018**	**0.008**	**0.01**	**0.10**
	No Info	Misinfo	-.054*	0.018	0.007	-0.10	-0.01
		Info	0.001	0.015	1.000	-0.03	0.04
	Info	Misinfo	-.055*	0.018	0.008	-0.10	-0.01
		No Info	-0.001	0.015	1.000	-0.04	0.03
Islam	Misinfo	No Info	0.071	0.030	0.053	0.00	0.14
		Info	**.138***	**0.031**	**0.000**	**0.06**	**0.21**
	No Info	Misinfo	-0.071	0.030	0.053	-0.14	0.00
		Info	.067	0.025	0.019	0.01	0.13
	Info	Misinfo	-.138*	0.031	0.000	-0.21	-0.06
		No Info	-.067	0.025	0.019	-0.13	-0.01
New Religions	Misinfo	No Info	**.127***	**0.034**	**0.001**	**0.04**	**0.21**
		Info	**.218***	**0.035**	**0.000**	**0.13**	**0.30**
	No Info	Misinfo	-.127*	0.034	0.001	-0.21	-0.04
		Info	**.091***	**0.028**	**0.004**	**0.02**	**0.16**
	Info	Misinfo	-.218*	0.035	0.000	-0.30	-0.13
		No Info	-.091*	0.028	0.004	-0.16	-0.02

*. The mean difference is significant at the 0.01 level.
