# Supplementary material for: Japanese Perception of Organ Donation and Implications for New Medical Technologies: Quantitative and Qualitative Social Media Analyses
Source: JMIR Form Res. 2024 Jul 19;8:e55797. doi: 10.2196/55797 (PMC11297371; doi:10.2196/55797)
Supplement: Multimedia Appendix 4 [file formative_v8i1e55797_app4.pdf]

Multimedia Appendix 4. Narratives related to organ donation.

**Actor**	**Narratives**	**Description**
Heart transplant doctor	Harvesting life[a]	
	Harvesting love[a]	
Medical students	Helping others[d]	
Other medical staff/Media	Profit[e]	Organ harvesting is done for profit.
Patient	Altruism[b]	Consists of prioritizing family in health decisions.
	お任せ(Omakase)[b]	To leave health decisions to their family.
	The patient looks alive[e]	The body is warm, the heart continues to beat, etc.
Patient relatives	頑張る (Ganbaru)[ab]	The patient is doing/did their best during their struggle with illness.
	五体満足(Gotai manzoku)[ab]	The wish to preserve the patient's body intact.
Japan Organ Transplantation Network	Connect life[c]	Through donation, the lives of several people are connected.
	Continuity of life[ac]	The patient continues to live in someone else.
	Gift of life[a]	
	Rebirth of life[a]	Also called "renewable life", donation gives new life to donor and recipient.
	Relay of life[ac]	The life of the patient passes on to others.
	Giving life[c]	A summary of all the other slogans by this actor.
Associations	Clarify donation intention[e]	Discuss the topic with relatives and use additional means besides a donor card to express donation intentions.
Religious actors	Individualized spirituality[e]	Death should not be determined by doctors and medical decisions should be made based on religious beliefs.
	Mechanistic view of life[e]	The body works like a computer and its parts can be used as spare parts.

[a] Based on [12]; [b] based on [18]; [c] based on [17]; [d] based on [20]; [e] authors
